# Competition between Heterochromatic Loci Allows the Abundance of the Silencing Protein, Sir4, to Regulate *de novo* Assembly of Heterochromatin

**DOI:** 10.1371/journal.pgen.1005425

**Published:** 2015-11-20

**Authors:** Michelle L. Larin, Katherine Harding, Elizabeth C. Williams, Noel Lianga, Carole Doré, Sophie Pilon, Éric Langis, Corey Yanofsky, Adam D. Rudner

**Affiliations:** Ottawa Institute of Systems Biology and Department of Biochemistry, Microbiology and Immunology, University of Ottawa, Ottawa, Ontario, Canada; University of California San Francisco, UNITED STATES

## Abstract

Changes in the locations and boundaries of heterochromatin are critical during development, and *de novo* assembly of silent chromatin in budding yeast is a well-studied model for how new sites of heterochromatin assemble. *De novo* assembly cannot occur in the G1 phase of the cell cycle and one to two divisions are needed for complete silent chromatin assembly and transcriptional repression. Mutation of *DOT1*, the histone H3 lysine 79 (K79) methyltransferase, and *SET1*, the histone H3 lysine 4 (K4) methyltransferase, speed *de novo* assembly. These observations have led to the model that regulated demethylation of histones may be a mechanism for how cells control the establishment of heterochromatin. We find that the abundance of Sir4, a protein required for the assembly of silent chromatin, decreases dramatically during a G1 arrest and therefore tested if changing the levels of Sir4 would also alter the speed of *de novo* establishment. Halving the level of Sir4 slows heterochromatin establishment, while increasing Sir4 speeds establishment. *yku70Δ* and *ubp10Δ* cells also speed *de novo* assembly, and like *dot1*Δ cells have defects in subtelomeric silencing, suggesting that these mutants may indirectly speed *de novo* establishment by liberating Sir4 from telomeres. Deleting *RIF1* and *RIF2*, which suppresses the subtelomeric silencing defects in these mutants, rescues the advanced *de novo* establishment in *yku70*Δ and *ubp10*Δ cells, but not in *dot1Δ* cells, suggesting that *YKU70* and *UBP10* regulate Sir4 availability by modulating subtelomeric silencing, while *DOT1* functions directly to regulate establishment. Our data support a model whereby the demethylation of histone H3 K79 and changes in Sir4 abundance and availability define two rate-limiting steps that regulate *de novo* assembly of heterochromatin.

## Author Summary

Heterochromatin, characterized by the repression of transcription, is a specialized chromatin structure that plays both structural and functional roles on chromosomes. Heterochromatic domains are dynamic, switching between active and inactive states, and this property is used by cells during developmental switches and may generate phenotypic diversity. We have shown that competition between different heterochromatic domains for limiting amounts of a heterochromatin protein, Sir4, plays a critical role in the switch from an active to an inactive state. Previous work has suggested that this switch is regulated by turnover of histone modifications in these regions and our data suggests that modulating Sir4 abundance acts in parallel to these changes to influence the rate of *de novo* assembly. This work supports a model in which competition between different chromosomal domains is exploited by cells to regulate cell identity.

## Introduction

Heterochromatin, or silent chromatin, is a specialized chromatin structure that plays structural and functional roles on chromosomes. These silent domains are characterized by repression of transcription and recombination, repressive histone modifications, and epigenetic inheritance [1]. The locations and boundaries of heterochromatic loci are dynamic and changing the landscapes of heterochromatin can cause changes in cell identity.

In the budding yeast, *Saccharomyces cerevisiae*, heterochromatin is found at the silent mating loci (*HML*α and *HMRa*, or the *HM* loci), telomeres and the rDNA gene loci. At the *HM* loci and telomeres, heterochromatin contains hypoacetylated and demethylated nucleosomes that are bound by the SIR (Silent Information Regulator) complex, which consists of three proteins: Sir2, Sir3 and Sir4 [1,2]. Sir2 is the founding member of a conserved family of NAD-dependent protein deacetylases and creates the hypoacetylated domains of nucleosomes within heterochromatin [3–5]. Sir3 and Sir4 are histone-binding proteins that bind with high affinity to deacetylated and demethylated nucleosomes [6–8]. Mutation of any of these three *SIR* genes abolishes both telomeric and *HM* silencing [9–14]. Current models for silent chromatin assembly propose that after initial recruitment of the SIR complex to nucleation elements, called silencers, at the *HM* loci, or to a telomere, iterative rounds of histone deacetylation and SIR complex recruitment lead to the spreading of the SIR complex [15–17]. After SIR complex spreading, recent work has suggested a final maturation step that requires the demethylation of K79 on histone H3, which is needed to form functional silent chromatin and trigger transcriptional repression [18–20].

The *de novo* assembly of heterochromatin is best understood in budding yeast where there is a block to assembly in G1 phase, and where assembly requires passage through S phase and dissolution of sister chromatid cohesion [21–23]. The S-phase requirement does not depend on DNA replication, but instead depends on some other event that occurs during S phase [24,25]. Current models propose that this cell cycle dependence reflects a cell cycle dependent removal of both histone modifications and the histone H2A variant, Htz1, which are refractory to heterochromatin assembly, and most cells take two cell cycles to fully silence a new site [19,26–28]. Methylation on K4 or K79 of histone H3, catalyzed by the Set1 and Dot1 methyltransferases respectively, inhibit heterochromatin assembly [29–33] and *de novo* assembly of heterochromatin occurs faster in *dot1Δ* or *set1Δ* mutants, supporting the model that these modifications must be removed before a new site of heterochromatin can assemble [19,26,34]. To date, however, there is no evidence indicating that methylation in heterochromatic regions is removed at specific points in the cell cycle.

Although changes in histone modifications are thought to regulate *de novo* assembly of silent chromatin, past work proposed that Sir4 may also play a role in regulating establishment. Silencing is sensitive to the dosage of Sir4: cells containing additional *SIR4* have improved silencing and heterozygous *SIR4/sir4Δ* diploids de-repress a weakened *HMR* locus [35]. These data led to the idea that the abundance of Sir4 may regulate the establishment of silencing, but the assays employed in these studies were not able to differentiate between defects in the establishment, maintenance, or stability of heterochromatin, and there is currently no evidence that Sir4 protein levels fluctuate.

We observed that Sir4 levels fall precipitously during a prolonged G1 arrest, and upon release from this arrest, Sir4 protein levels recover after two cell cycles, similar to the time required for *de novo* establishment of heterochromatin [19,21,26]. We therefore revisited the question of whether Sir4 dosage regulates *de novo* establishment using a single cell silencing establishment assay that monitors *HML*α repression directly [26]. We find that increasing Sir4 abundance speeds, and decreasing Sir4 abundance slows, the *de novo* establishment of heterochromatin. *ubp10Δ* and *yku70Δ* mutants, like *dot1Δ*, also speed *de novo* establishment as well as disrupt telomeric silencing. To investigate the relationship between *DOT1*, *UBP10*, *YKU70* and *SIR4*, we examined the speed of *de novo* establishment in *dot1Δ*, *ubp10Δ* and *yku70* mutants in conditions that lower Sir4 protein levels or that suppress their telomeric silencing defects. We conclude that *UBP10* and *YKU70* indirectly influence establishment by liberating Sir4 from telomeres, while *DOT1* acts directly at *HML*α, and propose a model in which the competition between telomeres and *HML*α allows limiting Sir4 levels to regulate *de novo* establishment.

## Results

### Sir4 protein decreases during a G1 arrest

**Fig 1:**
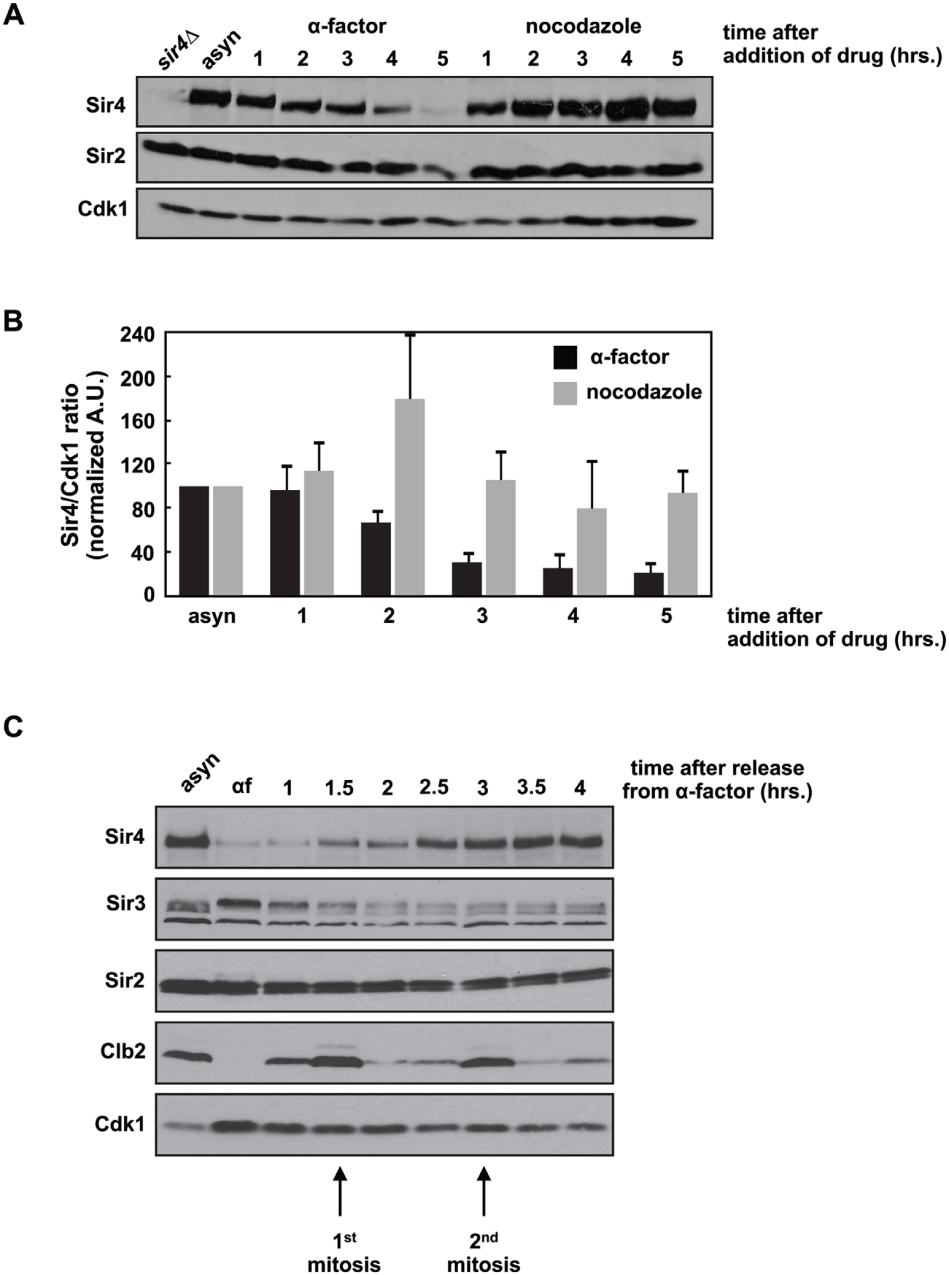
Sir4 protein is degraded during a prolonged arrest in G1 and takes two cell cycles to recover after release from the arrest. *(A)* Asynchronously growing (asyn) wild type (ADR4006) cells were arrested in G1 with 1μg/ml α-factor or arrested in mitosis with 10μg/ml nocodazole at 25°C. Samples were harvested every hour and protein levels were analyzed by western blot. Cdk1 is shown as a loading control. *(B)* Samples prepared as in *(A)* were quantified and the average amount of Sir4 (+/- SEM) relative to Cdk1 levels, normalized to the asynchronous (asyn) sample (which was arbitrarily set to 100), of three independent experiments is shown. *(C)* Asynchronously growing (asyn) wild type (ADR4006) cells were arrested in 1μg/ml α-factor for five hours (αf) and released from the arrest into fresh media at 25°C. Samples were harvested every 30 minutes and protein levels were analyzed by western blot. Cdk1 is shown as a loading control, and the mitotic cyclin, Clb2, is used as a marker of mitosis.

We were interested in whether Sir4 protein levels fluctuate during the cell cycle and observed that during a prolonged G1 arrest induced by the mating pheromone, alpha factor, Sir4 levels fell precipitously (Fig 1A). This decrease is not simply caused by cell cycle arrest, as cells arrested in mitosis with nocodazole maintain Sir4 levels (Fig 1A). Quantification of this experiment shows that Sir4 levels fall four- to five-fold after five hours of growth in alpha factor, but remain unchanged in nocodazole (Fig 1B). When pheromone-arrested cells are released back into the cell cycle, Sir4 protein levels recover after two cell cycles (Fig 1C). We also observed a similar drop in Sir4 protein levels when cells are arrested in stationary phase by prolonged growth in raffinose, a poor carbon source (S1A Fig). Re-feeding with dextrose allowed cells to re-enter the cell cycle and Sir4 levels recover after six hours.

This dramatic decrease in Sir4 abundance during a pheromone arrest has not been reported previously, and is paradoxical: Sir4, and the entire SIR complex, is required to maintain mating type identity, and mating type identity is needed to maintain a pheromone arrest. Sir4 localization by ChIP to a *HML*α silencer (*HML-E*) and *TELVI-R*, however, is not significantly different in cells arrested in G1 by pheromone, in mitosis by nocodazole, or grown asynchronously, confirming that existing sites of heterochromatin are maintained despite falling Sir4 protein levels (S1B Fig). In addition, the average intensity of intra-nuclear Sir4-GFP foci, which have previously been shown to mark clusters of telomeres and *HM* loci [36,37], are similar between cells arrested in G1 and mitosis (S1C and S1D Fig). Finally, most Sir4 is present on chromatin, and although there is less Sir4 in pheromone arrested cells, the ratio of chromatin to soluble Sir4 is similar in pheromone and nocodazole arrested cells (S1E Fig).

### *SIR4* is haploinsufficient for *de novo* establishment of silencing

Sir4 protein re-accumulates in two cell cycles after release from G1 arrest (Fig 1C). Previous reports have described similar timing for the establishment of *de novo* silencing [19,21,22,26], so we considered the possibility that Sir4 abundance may play a role regulating *de novo* establishment.

**Fig 2:**
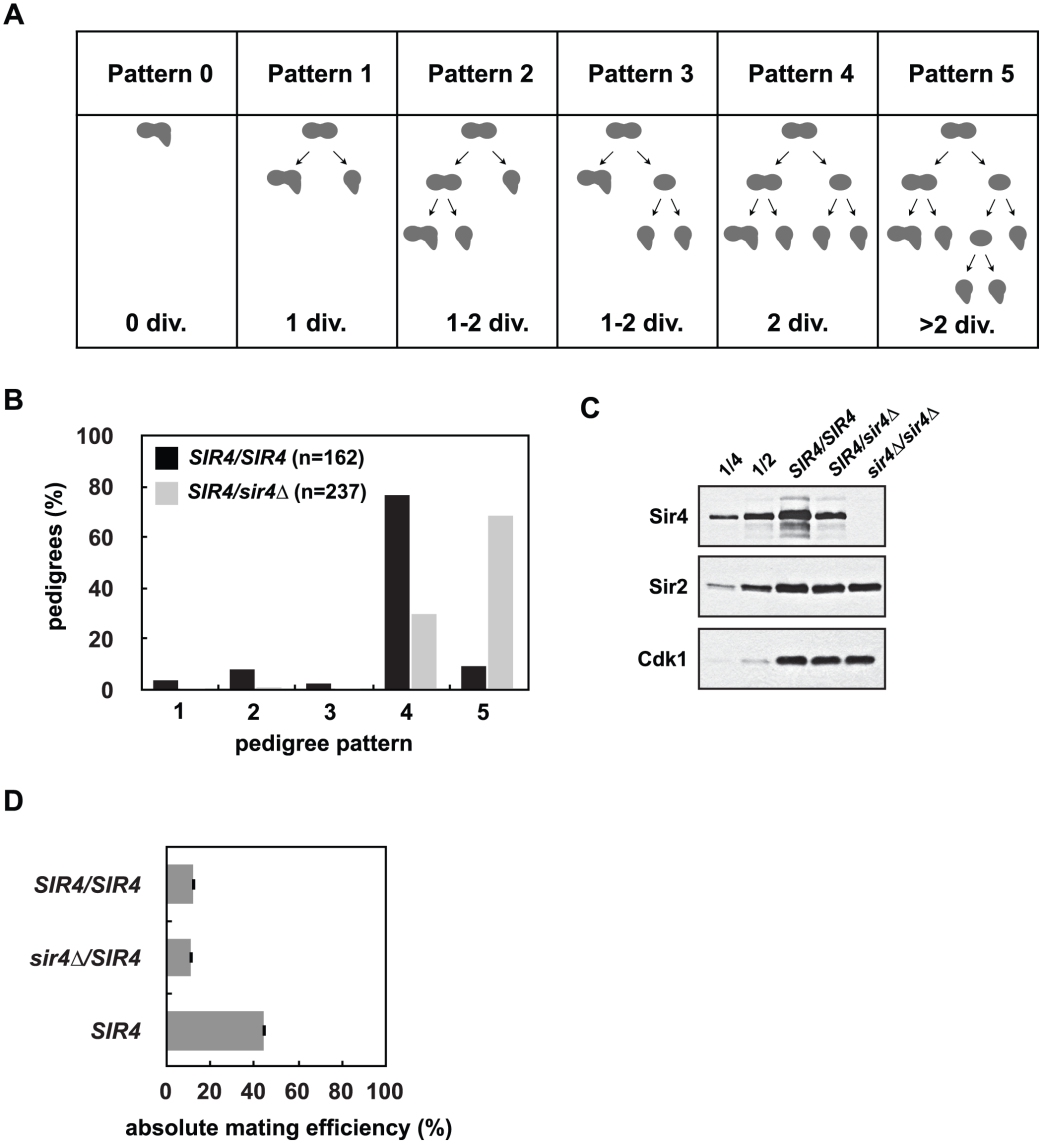
Reduced Sir4 abundance causes delays in *de novo* establishment of heterochromatin. *(A) De novo* establishment was monitored in a single cell establishment assay as described in Osborne *et al*. [26]. Specialized mating strains (JRY8828 and JRY8829) are mated and the behavior of the zygote and its progeny are grown adjacent to a large patch of *MAT*α cells (ADR22) and their response is monitored in a pedigree assay. JRY8828 has no mating information and will mate as an *MATa* cell. JRY8829, which has an intact *HML*α, but is also *sir3Δ*, will de-repress *HML*α and mate as an α cell. Immediately after mating the zygote will continue to mate as an *MAT*α cell because no other mating information is present. The zygote, however, is *SIR3/sir3Δ* and as soon as *HML*α is repressed, the zygote and its progeny will behave as a *MATa* cell and respond to α-factor pheromone which causes cell cycle arrest and the formation of a mating projection. The behavior of the zygote and its progeny are grouped into six pedigree patterns. Pattern 0 denotes a pedigree that silence *HML*α without dividing, Pattern 1 silence after one division, Pattern 4 after two divisions, and in Patterns 2 and 3 silencing is asymmetric—either the mother or first daughter silence after two divisions. Pattern 5 encompasses all pedigrees that silence after more than two divisions, including those that contain cells that don’t silence within the experiment. *(B)* Haploid cells that were either *SIR4* or *sir4*Δ were mated to form *SIR4/SIR4* (JRY8828 X JRY8829) and *sir4Δ/SIR4* (ADR4592 X JRY8829 or JRY8828 X ADR4593, see S2B Fig) diploid zygotes and were monitored for establishment of silencing at *HMLα* and categorized as in *(A)*. The effect of halving the Sir4 levels is significant (p<0.0000001, by the likelihood ratio test). *(C) SIR4/SIR4* (JRY8828 X JRY8829), *sir4Δ/SIR4* (ADR4592 X JRY8829), and *sir4Δ/sir4Δ* (ADR4592 X ADR4593) cells were grown at 25°C, harvested and analyzed by western blot. Two-fold serial dilutions of the *SIR4/SIR4* sample was analyzed to assess Sir4 concentration. *(D)* Quantitative mating assays were performed by crossing diploid strains from *(B)* and a wild type *MATa* strain (ADR21) to a *MATα* tester strain (ADR3082). The absolute mating efficiency is the proportion of cells of each query strain that mated and formed colonies on synthetic media lacking amino acids. There is no statistical significance between the mating efficiencies of *SIR4/SIR4* and *sir4Δ/SIR4* cells (Student’s two tailed t-test).

Previous work has shown that *SIR4* is haploinsufficient for silencing at a weakened and modified *HMRa (hmrΔA*::*ADE2*) [35], and we see a similar defect at a telomere proximal *URA3* reporter gene (*TELVII*-*L*-*URA3)* in *SIR4/sir4Δ* heterozygotes (S2A Fig). We wanted to determine whether this haploinsufficiency is caused by a defect in establishment or stability of heterochromatin, so we utilized a single cell silencing establishment assay in which two engineered strains are mated and the assembly of heterochromatin at *HML*α is monitored in the resulting zygote and its progeny (see [26] and Fig 2A for additional details). The diploid cells initially behave phenotypically as *MATα* cells, but upon silencing of *HML*α they switch identity and behave as *MATa* cells. This switch is monitored by the response of the diploids and their progeny to exogenous mating pheromone, which causes cell cycle arrest and polarization in cells that silence *HML*α. The number of cell divisions required for silencing establishment is determined by pedigree analysis (Fig 2A).

In this assay, approximately 80% of *SIR4/SIR4* homozygotes establish silencing at *HMLα* in two cell cycles ([26] and Fig 2B). *sir4*Δ*/SIR4* heterozygotes, however, establish silencing at *HML*α significantly more slowly (Fig 2B). Heterozygous *sir4Δ/SIR4* cells contain approximately half the amount of Sir4 as homozygous *SIR4/SIR4* cells (Fig 2C) and the establishment defect is independent of which strain is deleted for *SIR4* (S2B Fig). Once *HML*α is silenced (and the diploid zygotes mate as *MATa* cells), there is no significant difference in the mating efficiency of *SIR4/sir4Δ* versus *SIR4/SIR4* diploids (Fig 2D) indicating the defect in *sir4Δ/SIR4* cells is specific to silent chromatin establishment and not long-term stability. The pseudo-haploid diploids, however, mate with 3-fold lower efficiency than control haploid *MATa* cells (Fig 2D), perhaps due to an increase in size and ploidy compared to the control haploid cells.

### Increasing Sir4 levels speed *de novo* heterochromatin assembly

**Fig 3:**
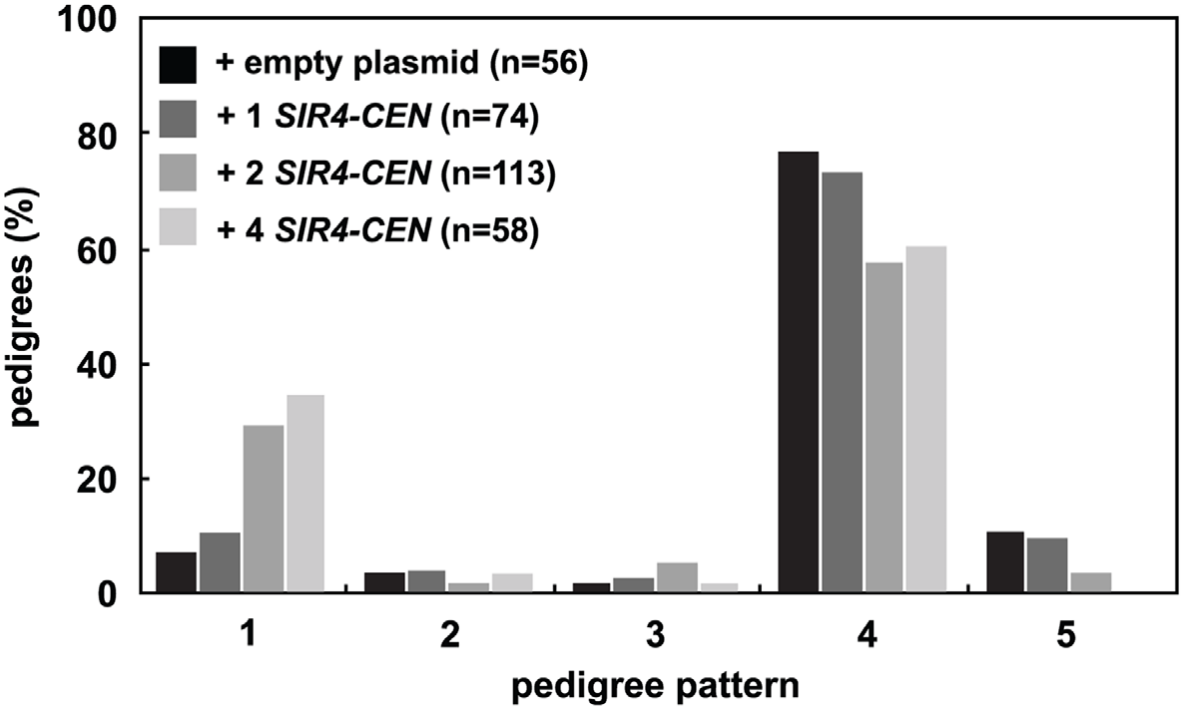
Increasing Sir4 speeds *de novo* establishment. One or two centromeric plasmids, each containing *SIR4* (pAR646 and pAR722), were transformed into one or both mating strains (JRY8828 and JRY8829) prior to mating. The empty plasmid control represents one empty centromeric plasmid in each strain (pRS313 or pRS316, and see S3C Fig). Cells were mated and the resulting zygotes were monitored and categorized as in Fig 2A. Addition of 2 *SIR4-CEN* plasmids or 4 *SIR4-CEN* plasmids were each significantly different from the empty plasmid distribution (p = 0.002 and p = 0.0004, respectively, by the likelihood ratio test). Statistics for every pairwise comparison can be found in S1 Table.

Because a decrease in the levels of Sir4 slows silent chromatin establishment, we wondered if increasing the concentration of Sir4 would speed establishment. Past work has shown that increasing Sir4 levels can improve silencing ([35] and S3A Fig), but has not distinguished whether this improvement is caused by improved stability or changes in the efficiency of establishment. Selection of one, two or four low-copy centromeric plasmids containing *SIR4* in zygotes increased the amount of Sir4 protein in these cells (S3B Fig) and significantly increased the speed of establishment in the single cell assay (Fig 3). These changes are not caused by the selection for multiple plasmids, as the presence of two or four empty plasmids does not change the speed of establishment as compared to cells containing no plasmids (S3C Fig).

Since the copy number of centromeric plasmids is variable in cells, we tested if other methods of increasing Sir4 would also improve silencing establishment. We created cells that contain *SIR4* driven by a galactose inducible promoter (*GAL-SIR4)* and constitutively express an estrogen receptor-Gal4 DNA binding domain fusion protein (*ER-GAL4-bd*) which allows graded expression of Sir4 using varying estradiol concentrations, rather than induction with galactose [38] (S3D Fig). The speed of silencing establishment in cells with increased Sir4 expression is increased, in a manner similar to that seen with the addition of the *SIR4-CEN* plasmids (S3E Fig).

Further increasing Sir4 levels, however, is detrimental to the establishment of silencing. High copy *2μ-SIR4* plasmids and strong overexpression of *SIR4* from a galactose inducible promoter (using galactose rather than estradiol) blocks or slows establishment, and causes derepression of both a telomeric *URA3* reporter and a weakened and modified *HMRa* (*hmrΔE*::*TRP1)* (S4 Fig). These findings are consistent with past data showing overabundance of Sir4 in cells disrupts silent chromatin by preventing assembly of a complete SIR complex [39–41].

### Epistasis analysis of *dot1Δ* and *SIR4/sir4Δ*

**Fig 4:**
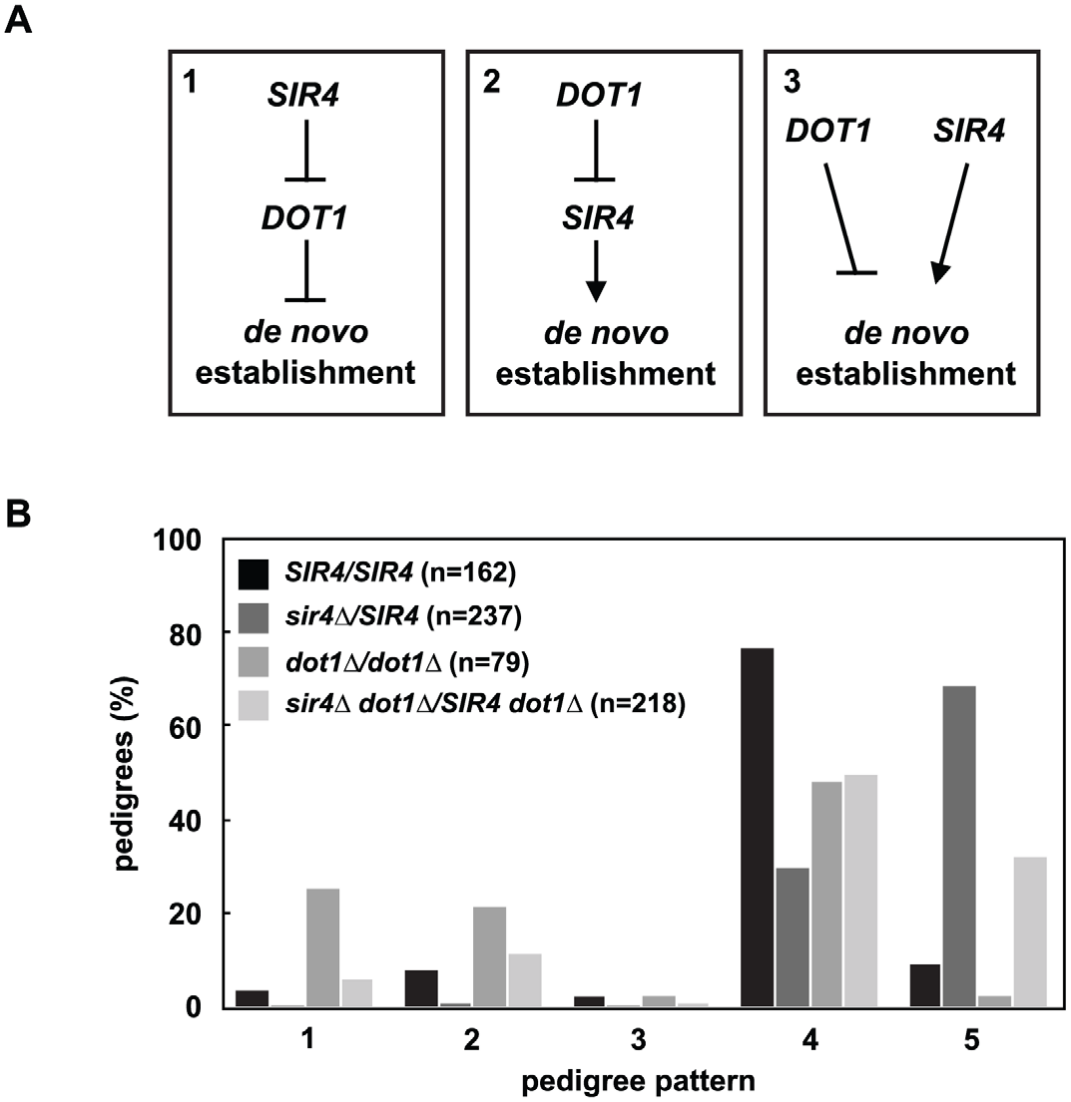
*DOT1* acts upstream or independently of Sir4 abundance. *(A) DOT1* and *SIR4* could regulate *de novo* establishment in one of three ways: Model 1) *SIR4* inhibits *DOT1*, and *DOT1* inhibits *de novo* establishment, model 2) *DOT1* inhibits *SIR4*, and *SIR4* promotes *de novo* establishment, and model 3) *DOT1* and *SIR4* function in separate pathways to regulate *de novo* establishment. *(B)* Cells were mated to create *SIR4/SIR4* (JRY8828 X JRY8829), *sir4Δ/SIR4* (ADR4592 X JRY8829), *dot1Δ/dot1Δ* (ADR4631 X ADR4632), and *sir4Δ dot1Δ/SIR4 dot1Δ* (ADR4631 X ADR5607 or ADR5640 X ADR4632), and the resulting zygotes were monitored and categorized as in Fig 2A. The *dot1Δ/dot1Δ* distribution is significantly different from the *sir4Δ dot1Δ*/*SIR4 dot1* and the *sir4Δ/SIR4* distributions (p<0.0000001 and p<0.0000001, respectively, by the likelihood ratio test). Statistics for every pairwise comparison can be found in S1 Table.

Deletion of *DOT1*, the histone H3 K79 methyltransferase, has also been shown to speed the *de novo* establishment of silent chromatin [19,26,34], which led to the proposal that the removal of histone H3 K79 methylation at silent loci may be a regulated step in *de novo* assembly. The effect of increasing Sir4 levels is similar to deleting *DOT1*, so we wondered if changes in Sir4 abundance acted upstream, downstream or independently of *DOT1* function (Fig 4A).

To test these three models we monitored silencing establishment in *dot1Δ SIR4/dot1Δ sir4Δ* diploids (Fig 4B). If Dot1 functions downstream of Sir4 abundance (Fig 4A, Model 1) then *dot1Δ SIR4/dot1Δ sir4Δ* diploids should be indistinguishable from *dot1Δ/dot1Δ* diploids. These diploids, however, are defective in the establishment of silent chromatin compared to *SIR4/SIR4* cells, although to a lesser extent than *SIR4/sir4Δ* cells. This result clearly rules out Model 1, in which Sir4 abundance functions upstream of Dot1, but this result alone cannot distinguish between the two remaining models (Fig 4A, Models 2 and 3).

### Deletion of *UBP10* and *YKU70* disrupt telomeric silencing and cause earlier establishment at *HML*α

In order to distinguish between the two remaining models (Fig 4A) we first considered how Dot1, a histone modifying enzyme, might act upstream of Sir4 abundance. Although the positive effect of *dot1Δ* on silencing establishment has been proposed to reflect changes in chromatin state at the *HM* loci [19,26], *dot1Δ* cells are also defective for subtelomeric silencing ([32,33] and S5A Fig), and this phenotype might indirectly affect the *HM* loci. Past studies have shown that telomeres and the *HM* loci compete for silencing proteins [42,43], and that loss of telomeric silencing can have indirect effects on other phenotypes due to re-localization of silencing proteins [44,45].

**Fig 5:**
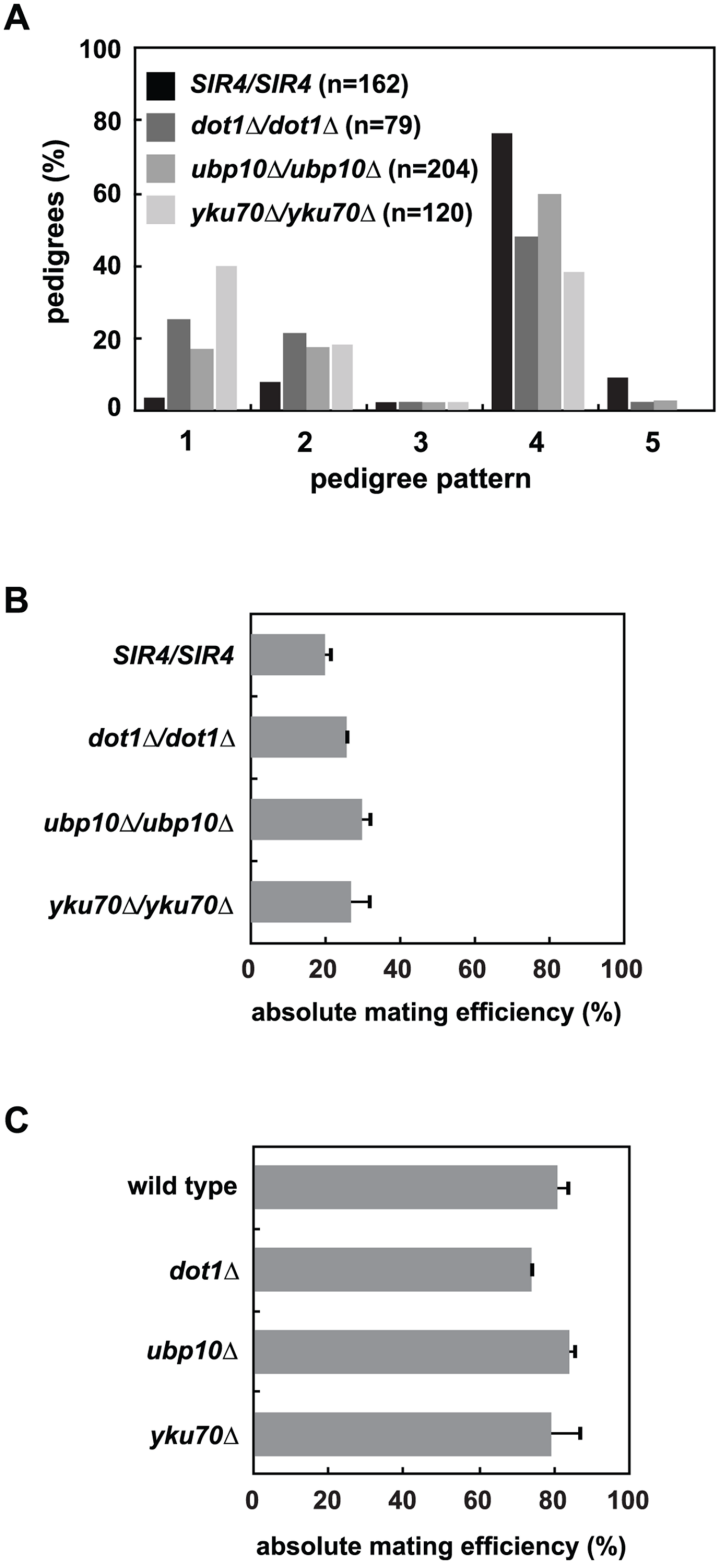
Mutation of *UBP10* and *YKU70* accelerate *de novo* establishment of heterochromatin. *(A) SIR4* (JRY8828 and JRY8829), *dot1Δ* (ADR4631 and ADR4632), *ubp10Δ* (ADR5087 and ADR5088) or *yku70Δ* (ADR5841 and ADR5842) cells were mated to produce homozygous zygotes that were monitored and categorized as in Fig 2A. *dot1Δ/dot1Δ* distribution is similar to experiments published by Osborne *et al*. [26]. All homozygous deletions are significantly different from the *SIR4/SIR4* control (p<0.0000001, by the likelihood ratio test). *(B)* Resultant diploids from *(A)* were mated to a *MATα* tester strain (ADR3082) to determine their mating efficiency. The absolute mating efficiency is the proportion of cells of each query strain that mated and formed colonies on synthetic media lacking amino acids. The average and SEM of at least three independent matings are graphed. There is no statistical significance between the mating efficiencies of the four strains (Student’s two tailed t-test). *(C)* Haploid *MATα* wild type (ADR22), *dot1Δ* (ADR6181), *ubp10Δ* (ADR6182) and *yku70Δ* (ADR6183) were mated to a *MATa* tester strain (ADR3081) to determine their mating efficiency. The average and SEM of at least three independent matings are graphed. There is no statistical significance between the mating efficiencies of the four strains (Student’s two tailed t-test).

Mutation of *UBP10*, a ubiquitin protease that targets K123 on histone H2B, allowed us to examine these two possible functions of Dot1 in silencing establishment. Unlike *dot1Δ* cells, deletion of *UBP10* increases Dot1-dependent K79 and Set1-dependent K4 methylation on histone H3 by increasing K123 ubiquitination on histone H2B ([46,47] and S6 Fig). However, similar to *dot1Δ* cells, *ubp10Δ* cells exhibit subtelomeric silencing defects detectable in strains containing a telomeric *URA3* reporter ([48] and S5A Fig). Interestingly, we also find that *ubp10Δ/ubp10Δ* cells, like *dot1Δ/dot1Δ* cells, speed the rate of establishment (Fig 5A).

To further test if loss of subtelomeric silencing could cause an earlier establishment phenotype at *HML*α, we deleted *YKU70*, a component of the Ku complex that is required for non-homologous end joining, normal telomere length maintenance and telomeric silencing [49,50], but is not required for silencing of the native *HM* loci (Fig 5B and 5C and [51–53]). Like *dot1Δ/dot1Δ* cells, *yku70Δ/yku70Δ* cells show a dramatic increase in the speed of silencing establishment (Fig 5A). Deletion of *YKU70*, *UBP10* or *DOT1* are specific to silencing establishment because the respective homozygous mutants have no effect on the long-term stability of *HML*α silencing in pseudo-*MATa* diploids (Fig 5B) or *HMRa* silencing in *MAT*α haploids (Fig 5C), similar to the *SIR4/sir4Δ* heterozygote (Fig 2D). Deletion of *UBP10* or *YKU70* also has no significant effect on K79 methylation of histone H3 at *HML*α relative to wild type cells ([46,47] and S6 Fig), demonstrating their effect on *de novo* silencing establishment is not due to inhibition of Dot1.

Like *dot1Δ/dot1Δ* cells, removing one copy of *SIR4* in *ubp10Δ/ubp10Δ* or *yku70Δ/yku70Δ* cells slows the speed of establishment compared to *SIR4/SIR4* cells (S7A and S7B Fig), which resembles the phenotype of a *SIR4/sir4Δ* heterozygote. This supports the hypothesis that all three of these proteins may speed *de novo* establishment indirectly by derepressing subtelomeric silencing. Double mutant diploids that combine *dot1Δ*, *ubp10Δ* and *yku70Δ* were also tested for epistatic effects using the single cell establishment assay. None of the pairwise deletions further increased the speed of establishment compared to the more penetrant single mutant within the pair (S7C–S7E Fig).

### Deletion of *RIF1* and *RIF2* suppress telomeric silencing defects in *yku70Δ*, *dot1Δ* and *ubp10Δ*, but have differing effects on the rate of *de novo* establishment

**Fig 6:**
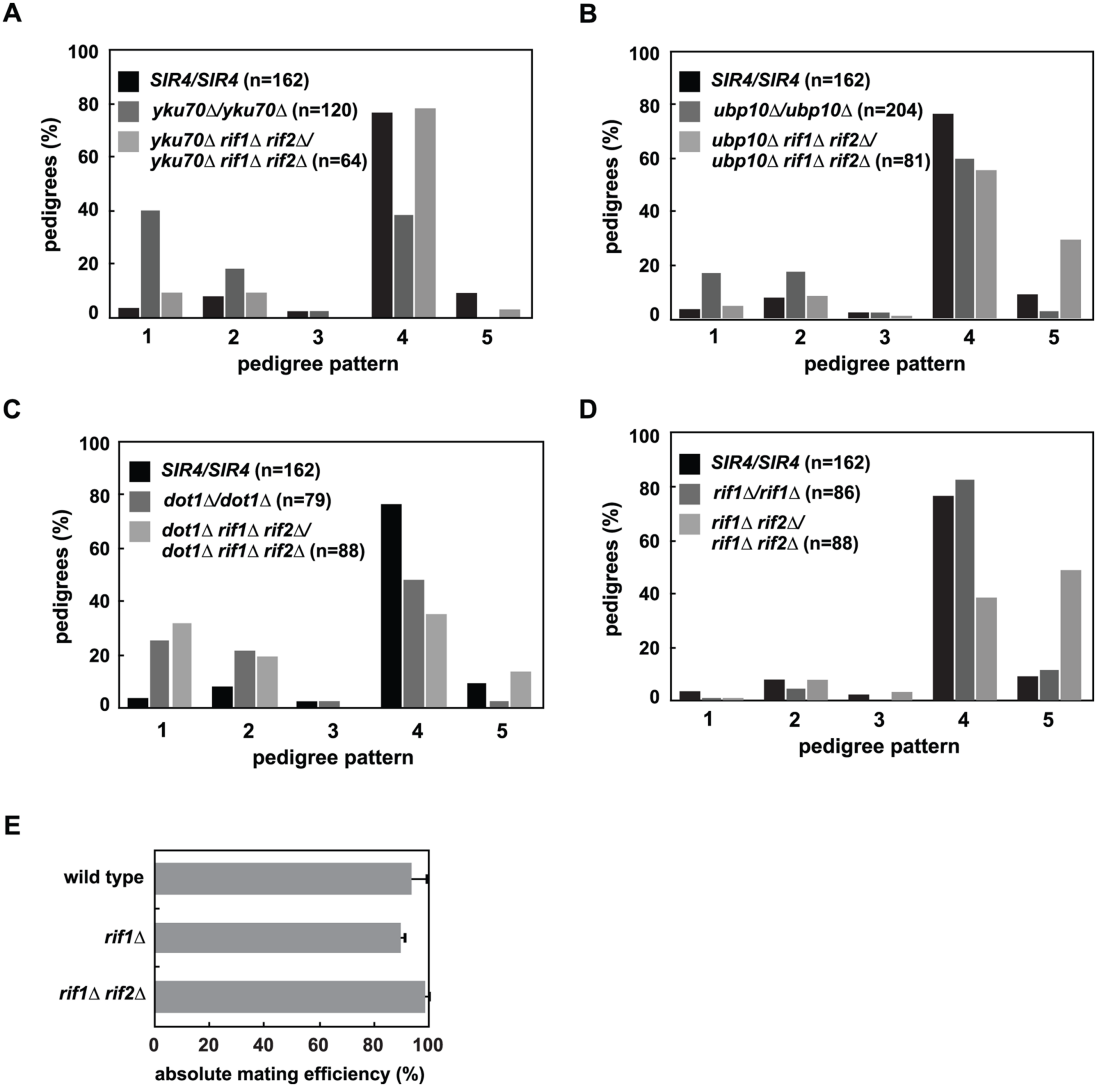
Mutation of *RIF1* and *RIF2* define two independent rate limiting steps in *de novo* establishment of heterochromatin. *(A) SIR4* (JRY8828 and JRY8829), *yku70Δ* (ADR5841 and ADR5842), *yku70Δ rif1Δ rif2Δ* (ADR7986 and ADR7989), or *rif1Δ* (ADR7962 and ADR7966) cells were mated to produce homozygous zygotes that were monitored and categorized as in Fig 2A. The distribution of *yku70Δ rif1Δ rif2Δ/yku70Δ rif1Δ rif2Δ* and *SIR4/SIR4* diploids are similar and *yku70Δ/yku70Δ* is significantly different from *yku70Δ rif1Δ rif2Δ/yku70Δ rif1Δ rif2Δ* diploids (p<0.0000001, by the likelihood ratio test). Statistics for every pairwise comparison of *A-D* can be found in S1 Table. *(B) SIR4* (JRY8828 and JRY8829), *ubp10Δ* (ADR5087 and ADR5088), and *ubp10Δ rif1Δ rif2Δ* (ADR8922 and ADR8888) were mated to produce homozygous zygotes that were monitored and categorized as in Fig 2A. The distribution of *ubp10Δ rif1Δ rif2Δ/ubp10Δ rif1Δ rif2Δ* and *SIR4/SIR4* diploids are similar and *ubp10Δ/ubp10Δ* is significantly different from *ubp10Δ rif1Δ rif2Δ/ubp10Δ rif1Δ rif2Δ* diploids (p = 0.00006, by the likelihood ratio test). *(C) SIR4* (JRY8828 and JRY8829), *dot1Δ* (ADR4631 and ADR4632), and *dot1Δ rif1Δ rif2Δ* (ADR8916 and ADR8913) cells were mated to produce homozygous zygotes that were monitored and categorized as in Fig 2A. The distribution of *dot1Δ/dot1Δ* and *dot1Δ rif1Δ rif2Δ/dot1Δ rif1Δ rif2Δ* and *SIR4/SIR4* diploids are similar and *SIR4/SIR4* is significantly different from *dot1Δ rif1Δ rif2Δ/dot1Δ rif1Δ rif2Δ* diploids (p<0.0000001, by the likelihood ratio test). *(D) SIR4* (JRY8828 and JRY8829), *rif1Δ* (ADR7962 and ADR7966), and *rif1Δ rif2Δ* (ADR9190 and ADR9196) cells were mated to produce homozygous zygotes that were monitored and categorized as in Fig 2A. The distribution of *SIR4/SIR4* and *rif1Δ/rif1Δ* cells are not significantly different from one another, and both are significantly different from *rif1Δ rif2Δ/rif1Δ rif2Δ* diploids (p<0.0000001, by the likelihood ratio test). *(E)* Haploid *MATα* wild type (ADR22), *rif1Δ* (ADR8824), and *rif1Δ rif2Δ* (ADR8833) were mated to a *MATa* tester strain (ADR3081) to determine their mating efficiency. The average and SEM of at least three independent matings are graphed. There is no statistical significance between the mating efficiencies of the three strains (Student’s two tailed t-test).

Deletion of the telomere binding proteins *RIF1* and *RIF2* suppress the subtelomeric silencing defect in *yku70Δ* cells ([54] and S5B Fig). This suppression allowed us to test if the advanced *de novo* establishment phenotype of *yku70Δ* cells at *HML*α depends on their subtelomeric silencing defect. We find that *rif1Δ/rif1Δ* and *rif1Δ rif2Δ/rif1Δ rif2Δ* mutants also suppress the earlier silencing establishment phenotype of *yku70Δ/yku70Δ* (Fig 6A and S7E Fig), supporting a model whereby effects on telomeric silencing in *yku70Δ* cells indirectly modulate the speed of silencing establishment at *HMLα* and act upstream of Sir4 availability (Fig 4A, Model 2).

We also find that deletion of *RIF1* and *RIF2* suppress the telomeric silencing defects of *dot1Δ* and *ubp10Δ* cells (S5B Fig), allowing us to rigorously test whether there is a correlation between the speed of silencing establishment at *HMLα* and the strength of subtelomeric silencing. As in *yku70Δ/yku70Δ* cells, *rif1Δ rif2Δ/rif1Δ rif2Δ* cells also suppress the earlier silencing establishment phenotype of *ubp10Δ/ubp10Δ* cells (Fig 6B). This suppression, however, is not seen in *dot1Δ/dot1Δ* cells (Fig 6C), despite the complete rescue of subtelomeric silencing in *rif1Δ rif2Δ dot1Δ* cells (S5B Fig), suggesting two distinct pathways operate to regulate *de novo* establishment of heterochromatin.

*rif1Δ rif2Δ* cells have extremely long telomeres which recruit large amounts of the telomere binding protein Rap1 [43,55,56]. Rap1, which also binds at the silencer elements of *HMR* and *HML*, binds to both Sir4 and Sir3, and is required for the nucleation of silent chromatin [57–60]. The increased recruitment of Rap1 to telomeres in *rif1Δ rif2Δ* cells has been speculated to cause the loss of silencing at weakened *hmr* silencers ([43,55] and S5B Fig), suggesting that the suppression of the subtelomeric silencing defects of *dot1Δ*, *ubp10Δ* and *yku70Δ* cells is also caused by increased recruitment of Sir4 and Sir3 to telomeres. Consistent with this model and our hypothesis that the release of subtelomeric Sir4 speeds *de novo* establishment in *yku70Δ/yku70Δ* and *ubp10Δ/ubp10Δ* cells, we find that *rif1Δ rif2Δ/rif1Δ rif2Δ* cells have the opposite phenotype and slow the *de novo* establishment of silencing in a manner similar to that of *SIR4/sir4Δ* cells (Fig 6D). Like *SIR4/sir4Δ* cells (Fig 2D), *rif1Δ* and *rif1Δ rif2Δ MATα* haploids have similar mating efficiency as wild type cells (Fig 6E), indicating that once silencing is established at *HMLα*, these mutants have no defects in maintaining the silent state.

## Discussion

### Changes in Sir4 abundance regulate *de novo* assembly of silent chromatin

We observed that Sir4 protein levels fall during a prolonged arrest in G1 and re-accumulate over two cell cycles after release from this arrest (Fig 1). This is the first report of cell cycle-dependent changes in Sir protein abundance, and prompted us to directly test if Sir4 abundance modulates the *de novo* assembly of silent chromatin. Using a single cell establishment assay we have shown that decreasing Sir4 dosage in a *SIR4/sir4Δ* heterozygote slows *de novo* assembly at *HMLα* (Fig 2), and increasing Sir4 dosage speeds assembly (Fig 3). Importantly, the phenotype of *SIR4/sir4Δ* cells is specific to establishment, as a quantitative mating assay shows that there is no defect in the long-term stability of silencing at *HML*α (Fig 2B). These results suggest that in the single-cell pedigree assay the accumulation of Sir4 is a rate-limiting step in silencing establishment.

The original experiments that suggested Sir4 dosage might regulate establishment used an *ADE2* reporter placed at a weakened and modified *HMRa* locus (*hmrΔA*-*ADE2*) and monitored repression and/or activation of *ADE2* by red/white colony sectoring [35]. Although this assay was proposed to monitor establishment of silent chromatin, changes in sectoring may also monitor the maintenance or stability of heterochromatin. We tested if this sectoring assay could be used as a simpler alternative to the single-cell pedigree assay, and found that *dot1Δ* and *yku70Δ* cells, which speed establishment in the pedigree assay (Fig 5A), derepress *ADE2* (S5A Fig). Thus, in the context of a weakened silencer at *HMR*, these mutants negatively impact silencing. We conclude that although experiments using *hmrΔA*::*ADE2* strains identified Sir4 as a dose-dependent regulator of silencing [35], this assay may not always reflect changes in *de novo* establishment.

### Sir4 abundance acts in parallel to changes in histone H3 K79 methylation

Past studies have shown that removal of histone H3 K4 and K79 methylation, and of Htz1-containing nucleosomes, which are all refractory to heterochromatin assembly, is rate-limiting for *de novo* silencing establishment [19,26–28,34]. We therefore wondered whether Dot1-dependent histone H3 K79 methylation acted in the same pathway as *SIR4*, or in an independent pathway.

The epistasis analysis between *dot1Δ/dot1Δ* and *SIR4/sir4Δ* cells clearly demonstrates that *DOT1* inhibition of establishment is not downstream of the effects of changes in Sir4 abundance (Fig 4A and 4B, Model 1). Our finding that *yku70Δ/yku70Δ* and *ubp10Δ/ubp10Δ* cells also speed establishment (Fig 5A) suggested a model whereby the loss of subtelomeric heterochromatin common to the *dot1Δ*, *yku70Δ* and *ubp10Δ* mutants [32,33,41,49] may indirectly cause faster establishment of silencing at *HMLα*.

Loss of one copy of *SIR4* in all three mutants causes an intermediate phenotype between *SIR4/SIR4* and *SIR4/sir4Δ* (Fig 4B and S7A and S7B Fig), so these experiments cannot differentiate between a model that these genes act upstream or independently of Sir4 abundance (Fig 4A, Models 2 and 3). An intermediate phenotype might be expected if these genes acted independently of Sir4 abundance. Similarly, because *SIR4/sir4Δ* is not a null mutant and still contains Sir4, some suppression of the *SIR4/sir4Δ* phenotype might be expected if these genes acted upstream of Sir4 abundance, and when mutated liberated Sir4 from telomeres.

**Fig 7:**
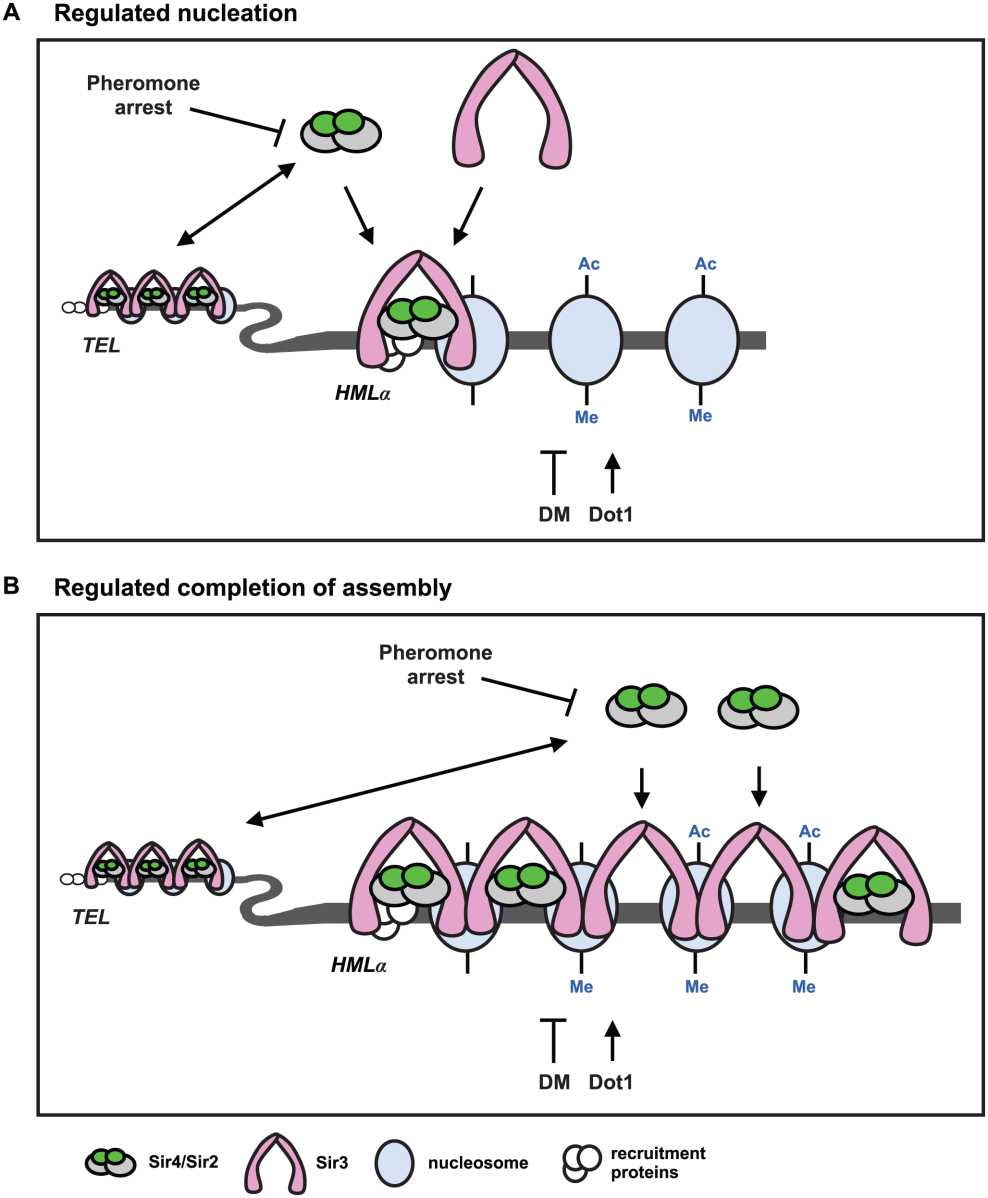
Two models for *de novo* establishment of heterochromatin. *(A) Regulated nucleation*. Changes in Sir4 availability and demethylation of histone H3 regulate nucleation of heterochromatin. The abundance and availability of Sir4 is downregulated during pheromone arrest, and telomeres and *HML*α compete for the available Sir4. When Sir4 is present in extra copies, or is released from telomeres in *ubp10Δ* or *yku70Δ* mutants, nucleation occurs faster. If the available Sir4 is reduced in heterozygous *SIR4/sir4Δ* cells or in *rif1Δ rif2Δ* cells, that improve telomeric recruitment of Sir4, nucleation slows. These data suggest that recruitment of Sir4 to the *HML*α silencer is rate limiting for *de novo* establishment of heterochromatin. Sir4/Sir2 recruitment leads to deacetylation (Ac) of proximal nucleosomes by Sir2, and promotes the recruitment of Sir3 which interacts with the deacetylated N-terminal tails of histone H4. Dot1 adds, and demethylation (DM) removes, methylation (Me) on lysine 79 of histone H3. Demethylation (DM) may occur enzymatically by an unidentified demethylase, by histone exchange, or by deposition of unmethylated histones after DNA replication. Sir3 interacts specifically with unmethylated histone H3, so removal of K79 methylation also promotes Sir3 binding to nucleosomes, and at silencer elements, both deacetylation and demethylation may be required for nucleation of heterochromatin. In *dot1Δ* mutants, that have no K79 methylation, *de novo* establishment occurs faster and suggests demethylation of K79 and subsequent recruitment of Sir3 can also be rate limiting for *de novo* establishment of heterochromatin. Coincident recruitment of Sir3 and Sir4, and their interaction, is required for efficient *de novo* establishment. *(B) Regulated completion of assembly*. Changes in Sir4 availability and demethylation of histone H3 regulate transcriptional repression. Kirchamaier and Rine [20] observed a rate limiting step in *de novo* establishment after spreading of Sir proteins at *HMRa*, and Katan-Khaykovich and Struhl [19] showed demethylation of K79 on histone H3 is a slow step in *de novo* establishment. If the abundance of Sir4 acted at a late step in establishment at *HMLα*, it would suggest that Sir4 occupancy and histone deacetylation may be incomplete in heterochromatin prior to demethylation of histone H3. Recruitment of Sir4 into silent chromatin would allow complete histone deacetylation and be mechanistically linked to histone demethylation and transcriptional repression.

Our analysis of how deletion of *RIF1* and *RIF2* interacts with these three mutants significantly clarified our conclusions and suggests that *DOT1* and Sir4 abundance function in independent pathways to regulate *de novo* establishment. Although *rif1Δ rif2Δ* cells suppress the subtelomeric silencing defects of *yku70Δ*, *ubp10Δ* and *dot1Δ* cells (S5B Fig and [54]), the earlier establishment phenotype is only rescued in *rif1Δ rif2Δ yku70Δ/rif1Δ rif2Δ yku70Δ* and *rif1Δ rif2Δ ubp10Δ/rif1Δ rif2Δ ubp10Δ* cells (Fig 6A and 6B). In contrast, *rif1Δ rif2Δ dot1Δ/rif1Δ rif2Δ dot1Δ* cells, like *dot1Δ/dot1Δ* cells, establish heterochromatin faster than wild type cells. These differences suggest that *DOT1* functions in a distinct pathway from *YKU70* and *UBP10* (Fig 7).

### A model for *de novo* establishment

Our data argue that either increased Sir4 or the absence of histone H3 K79 methylation speeds *de novo* establishment of heterochromatin. These two events could regulate a common step in heterochromatin assembly, or function independently of each other. Because we see similar phenotypes in cells with increased Sir4 as with mutation of *DOT1* and *YKU70*, and we don’t observe additive effects in *dot1Δ yku70Δ/dot1Δ yku70Δ* or *dot1Δ ubp10Δ/dot1Δ ubp10Δ* mutants (S7C–S7E Fig), we favour a model in which a common step in heterochromatin assembly is regulated by both Sir4 and histone H3 demethylation (Fig 7).

Past work has shown that recruitment of Sir4 to silencers and telomeres occurs independently of Sir2 and Sir3 [15–17,61] and this first step in the nucleation of silent chromatin may be slowed during periods of limiting Sir4 protein (Fig 7A, *regulated nucleation*). Dot1-dependent histone methylation directly antagonizes Sir3 binding to nucleosomes [62,63], so the absence of H3 K79 methylation in *dot1Δ* cells may also regulate heterochromatin nucleation by improving Sir3 recruitment to the *HML* silencer. In this model, coincident recruitment of Sir3 and Sir4, and their binding to one another, would be a slow step required for efficient nucleation (Fig 7A and [64]). Overexpression of Sir3 does not speed establishment [26], which is consistent with a model that Sir3 recruitment is determined by the extent of demethylation, not the concentration of Sir3. The extent of histone H3 K79 methylation at *HML-E* is similar (and low) in the presence or absence of *SIR4* (S6 Fig), suggesting that histone H3 K79 demethylation at *HML-E* may be regulated independently of heterochromatin assembly, and that this function of demethylation is unlikely to be a regulated step in *de novo* establishment.

Alternatively, because histone H3 K79 methylation often correlates with active transcription [65–67] and competes with Sir3 and Sir4 for binding to nucleosomes [31,68,69], Dot1 may control Sir3 spreading, which could also function as a rate limiting step in *de novo* assembly (Fig 7B, *regulated completion of assembly*). In this model, low levels of Sir4 would be sufficient for the spreading of Sir proteins, but additional Sir4 would be necessary at a later step, arguing that the occupancy of Sir4 would change during transcriptional repression of this region (Fig 7B). The recruitment of the Sir4/Sir2 complex could be linked to the demethylation of histone H3 K79, as previous work has shown that Dot1 competes with Sir3 and Sir4/Sir2 for binding to the N-terminal tail of histone H4 [31,68,69].

Supporting this model, several studies have suggested histone H3 K79 demethylation functions at a late step in silent chromatin maturation [18–20,70] and one showed that *de novo* establishment at a modified *HMRa* occurred after SIR complex recruitment and spreading [20]. This work investigated *de novo* establishment at *HMRa*, which occurs more slowly than at *HMLα* [71,72], and establishment was triggered by the tethering of several Sir1 proteins, which recruit both Sir3 and Sir4 [73,74]. Both aspects of this experimental design, therefore, may alter the requirements for Sir4 and histone H3 K79 demethylation.

Although histone H3 K79 demethylation speeds establishment at strong silencers, in the context of mutant silencers (*hmrΔE* or *hmrΔA*), *TELVII-L*, or the deletion of the recruitment protein Sir1, the absence of this methylation causes defects in silencing (S5 Fig and [26,31–33,41,75]). We speculate that these defects are likely caused by the inability of the weak silencers to compete effectively with the recruitment of Sir3 non-specifically to other genomic loci [32,33,70].

### Competition between heterochromatic sites regulates *de novo* establishment

Past work showing that telomeres act as reservoirs of Sir proteins [42,43,76–82] suggest a model in which the loss of subtelomeric silencing in *yku70Δ* and *ubp10Δ* cells liberates Sir4 from telomeres and increases its availability for *de novo* establishment at *HMLα* (Fig 7). In such a model the competition between telomeres and potential new sites of silencing would allow changes in Sir4 abundance to regulate the speed at which these new sites of heterochromatin can assemble.

*rif1Δ* and *rif1Δ rif2Δ* cells have been shown to antagonize silencing at weakened *HM* loci, likely due to increased recruitment of the SIR complex to telomeres, and like *sir4Δ/SIR4* cells, *rif1Δ rif2Δ/rif1Δ rif2Δ* cells slow *de novo* establishment (Fig 6D). These mutants provide independent support of our hypothesis that telomere sequestration of Sir4 regulates *de novo* establishment. The phenotypes of *rif1Δ rif2Δ/rif1Δ rif2Δ* cells are milder than *sir4Δ/SIR4* cells, which may explain why *yku70Δ sir4Δ/yku70Δ SIR4* cells establish silencing slower than *yku70Δ rif1Δ rif2Δ/yku70Δ rif1Δ rif2Δ* cells. We observe similar differences in the phenotype of *dot1Δ/dot1Δ* cells when combined with *sir4Δ/SIR4* or *rif1Δ rif2Δ/rif1Δ rif2Δ*, but in this situation we hypothesize that less Sir4 is needed for robust establishment at *HMLα* even though telomeric silencing is strengthened in *rif1Δ rif2Δ/rif1Δ rif2Δ* cells.

Competition between heterochromatic sites has also been described in fission yeast in which sequestration of Swi6 at telomeres regulates the efficiency of assembly of heterochromatin at other sites, and release of Swi6 from telomeres or increased Swi6 expression allows bypass of RNAi-dependent assembly of pericentric heterochromatin [83]. This work suggested that a function of telomeric heterochromatin is to buffer cells from changing levels of heterochromatin factors and prevent inappropriate assembly of potentially harmful heterochromatin. Our work supports this model, but also suggests that competition between telomeric heterochromatin could function to generate phenotypic diversity within a population. Sir-dependent regulation of subtelomeric genes has been shown to influence cell adhesion, cell wall remodeling and stress resistance in budding yeast, expression of cell surface antigens in *Plasmodium falciparum*, and colony morphologies in *Candida albicans* [84–89]. Some of these changes are induced by environmental factors, but variation in the expression of a heterochromatin protein, like Sir4, may also influence the competition between different subtelomeric regions to generate more subtle changes in cell identity.

### How and why is Sir4 abundance regulated?

Our investigation into the role of Sir4 in *de novo* establishment began with the surprising observation that Sir4 abundance falls precipitously after prolonged G1 arrest (Fig 1). The re-synthesis of Sir4 after release from this arrest takes two cell cycles, which correlates with the time needed for cells to establish a new site of heterochromatin [19,21,26]. Although we are unable to monitor Sir4 protein levels during mating in individual cells, we hypothesize that the prolonged exposure to pheromone during mating also causes a drop in Sir4 protein abundance. After mating, *SIR4/SIR4* zygotes would then require two cell cycles to re-synthesize Sir4 and re-establish silencing of *HMLα*.

The slow kinetics of Sir4 degradation may explain why this drop in abundance has not been observed previously, and suggests that the decrease in Sir4 protein levels may be induced by pheromone, and not cell cycle arrest. *SIR4* transcription is unchanged during pheromone treatment [90], thus the drop in protein level is likely caused by regulated translation or degradation. Recent work has defined a pheromone-induced pathway that slows cell growth by causing inhibition of ribosome synthesis and translation [91]. The strength of this response depended on the concentration of pheromone and the extent of polarization, thus we speculate that the slow drop in Sir4 over five hours of arrest (Fig 1) may indicate that Sir4 is regulated by the same pathway.

Our finding that the abundance and availability of Sir4 can regulate *de novo* heterochromatin establishment suggests that changes in Sir4 abundance may be a cell cycle regulated step in *de novo* establishment [21,22,24,25]. However, although we have shown that modulating Sir4 abundance changes the speed of establishment in single cells (Figs 1 and 2), we have not been able to test if preventing the loss of Sir4 in pheromone arrested cells will allow for immediate re-establishment during the arrest. Identifying the mechanism that regulates Sir4 abundance will allow us to test this directly.

Chromatin fractionation revealed that both the soluble and chromatin-bound fraction of Sir4 drops during G1 arrest (S1E Fig). An explanation for this behavior is that the majority of chromatin-bound Sir4 binds to non-specific chromosomal sites and that this fraction rapidly equilibrates with soluble Sir4, such that bound and unbound bulk levels of the protein drop equally during G1 arrest. Sir4 bound at specific chromosomal sites (S1B Fig), however, may not exchange as rapidly, allowing maintenance of silencing despite low levels of Sir4.

Although the level of Sir4 protein during a pheromone arrest may be insufficient to establish new sites of silent chromatin, past work has shown that existing subtelomeric silent chromatin are *more* stable during a pheromone arrest [92]. The stability of existing sites would imply that two factors are at play: low levels of Sir4 prevent new sites of heterochromatin from forming, and structural or post-translational changes in Sir4 (or another silencing protein) prevent disassembly of existing sites. Recent work has shown that very low levels of Sir3, like Sir4, are also sufficient to maintain a silenced state [93]. This strategy may be useful for cells arrested in G1, where maintenance of cell identity is critical as cells make developmental choices including the decision to mate, to initiate the meiotic program, to enter the cell cycle, or to enter quiescence. In addition, low levels of Sir4 have also been shown to improve silencing within the rDNA [82], providing a mechanism for cells to maintain rDNA integrity during persistent G1 arrest. Similar developmental decisions are made in vertebrate cells in G1, and similar mechanisms may be used to protect cell identity.

## Materials and Methods

### Ethics statement

This study was performed in strict accordance with standards for animal care and use outlined in the Canadian Council on Animal Care Standards. The University of Ottawa is a registered research facility under the Province of Ontario's Animals for Research Act. The protocol was approved by the University of Ottawa Animal Care Committee (Permit Number: BMI-113). All surgery was performed under sodium pentobarbital anesthesia, and every effort was made to minimize suffering.

### Strain and plasmid construction

Supporting information S2 Table lists the strains used in this work. All strains, except the mating testers (ADR3081 and ADR3082; gifts from Fred Winston, Harvard Medical School) are derivatives of the W303 strain background (W303-1a; Rodney Rothstein, Columbia University, New York, NY). Strains used for the single cell mating assay (JRY8828 and JRY8829) were a gift from Erin Osborne and Jasper Rine (UC, Berkeley, CA). All deletions and replacements were confirmed by immunoblotting, phenotype or PCR. The sequences of all primers used in this study are available upon request. The bacterial strains DH5α and Rosetta (EMD-Millipore) were used for amplification of DNA.

All deletions were made using cassettes amplified from pAR747 (*C*. *albicans URA3)*, pFA6a-His3MX6 (*S*. *pombe his5* ^*+*^), pFA6a-kanMX6 [94], pAG29 (*natMX4*) [95] and pYM22 (*K*. *lactis TRP1*) [96]. pAR747 was constructed by cloning the *CaURA3* gene from pKT176 [97] as a BglII/XmaI fragment into pAG29 (*patMX4*) [95] cut with BglII/XmaI.

The *TELVII-L*::*URA3* and *adh4*::*URA3* strains were made by using pTEL::URA3 and pADH4::URA3, respectively (kindly provided by Dan Gottschling, Fred Hutchison Cancer Research Center) to create ADR2828 and ADR2830, respectively.

*hmrΔE*::*TRP1* [35] strains were constructed by crossing derivatives of CCFY100 (kindly provided by Kurt W. Runge, The Lerner Research Institute, Cleveland, OH) [98] to create ADR4062 which was subsequently used to create other *hmrΔE*::*TRP1* strains.

*hmrΔA*::*ADE2* [35,99] strains (kindly provided by David Shore (University of Geneva, Switzerland) via Marc Gartenberg (Rutgers, NJ)) were constructed by deleting *DOT1*, *UBP10* and *YKU70* in GCY317.

The *SIR4-eGFP* strain was created using pKT127 [97]. *his3-11*,*15*::*pGAL-SIR4-HIS3* strains were created by integrating pAR655 cut with BsiWI into the appropriate strains. pAR655 was constructed by amplifying the entire *SIR4* ORF by PCR and cloning it as a ClaI/EcoR1 fragment into pAR121. pAR121 is pRS303 [100] with the *GAL1-10* promoter cloned between the KpnI/XhoI sites.

*leu2-3*,*112*::*pMRP7-GAL4-ER-VP16-LEU2* strains were created by integrating pAR941 cut with XcmI. pAR941 was created by cloning the pGEV cassette as a FspI fragment from pGEV-HIS3 [38] into pRS305 [100].

*SIR4-CEN* plasmids, pAR646 and pAR722 were created by cloning the XhoI/EcoR1 fragment that contains *SIR4* from pAR465 into pRS313 (*HIS3*) and pRS316 (*URA3*) [100] respectively. pAR465 is a modified version of LSD343 (pRS314-SIR4; kindly provided by David Shore) which includes 216 nt downstream of the STOP codon followed by a XhoI site and a silent MluI site has been introduced at nt2850 (changing AAGAGT to ACGCGT). pAR450 contains the same *SIR4* fragment as pAR465 (without the MluI site) cloned into pRS313 [100]. pRS313 and pRS316 [100] were used as empty *CEN* plasmids in pedigree experiments. The *SIR4-2μ* plasmid was created by cloning the XhoI/EcoR1 fragment that contains *SIR4* from pAR646 into pRS423 (*HIS3*) [100]. pRS423 was used as the empty *2μ* plasmid in pedigree experiments.

### Pedigree assay

The single cell establishment pedigree assay was performed as described in Osborne et al. [26] with minor variations. Cells were grown overnight on plates of either YEP + 2% Dextrose, YEP + 2% Raffinose or synthetic selective media at 30°C, with the exception of *ku70Δ dot1Δ* (ADR5920 and ADR5921) and *ku70Δ dot4Δ* (ADR5944 and ADR5945) cells which were grown at 25°C overnight due to a slight temperature sensitivity. A small number of cells of each experimental strain were resuspended in YEP and each spotted onto a YEP +2% dextrose plate along with a thick streak of *MAT*α (ADR22) cells. Individual cells from the two experimental strains were micro-manipulated (Nikon Eclipse 50i, 20X S Plan Fluor, NA0.45, with TDM50 micromanipulator) next to one another and allowed to mate over one to two hours. Zygotes were micro-manipulated adjacent to the streak of *MAT*α cells and allowed to grow at 30°C. As the zygote (and subsequent daughters) divided, daughters were separated and moved in order to observe and score the behavior of the pedigree. Cells were monitored every one hour by microscopy and tracked for three divisions or until all cells in the pedigree had arrested and shmooed. Similar to Osborne et al. [26], we did not observe plate-specific or day-specific effects on the pedigree patterns, and we therefore pooled data from several plates to compile the distributions for each genotype tested.

Statistical differences between pedigree distributions were determined by likelihood ratio test as described [26]. Each distribution was compared pair-wise, and the complete dataset can be found in Supporting Information S1 Table.

### Physiology and microscopy

Cell cycle arrests were performed with 10μg/mL nocodazole (Sigma-Aldrich), 1μg/mL α-factor (Biosynthesis) or 0.2M hydroxyurea (HU; Sigma) at 25°C.

To image Sir4 foci, *SIR4-GFP* and wild type (ADR4006) control cells were grown overnight in YEP + 2% dextrose to log phase, fixed in 4% paraformaldehyde for 10 minutes, washed, sonicated and resuspended in 100mM KPO_4_ containing 1.2M sorbitol. Samples were imaged using a Nikon TI microscope (Nikon) with a Nikon Plan Apo 60X 1.4 NA objective and FITC filter set (Chroma) at room temperature with a Photometrics CoolsnapHQ2 camera (Photometrics). 13 fluorescent images using no ND filters and an exposure time of 2s were obtained separated by 0.5μm along the Z-axis. A single brightfield image was obtained at the central plane with an exposure time of 200ms. Example images were prepared using ImageJ software. Imaging was done in mixed populations of nocodazole (mitotic) and α-factor (G1) treated cells; the two cell types were determined by cell morphology. The same linear look-up-table was used for each example image. Fluorescence quantification was done using NIS-Elements software. For each cell three fluorescent foci were analyzed. A total of eight cells of each genotype were obtained in two separate experiments. Mean fluorescence in a 0.25um^2^ circular ROI was obtained for each focus. For each focus, mean fluorescence from a similar sized background ROI obtained from the same cell and focal plane was subtracted. Non-focus fluorescence measurements were obtained as described above but all images were obtained at the same Z-plane. Three measurements were obtained in each of eight cells from two separate experiments.

### Silencing assays

For serial dilution assays, cells were grown for two days in YEP + 2% dextrose or synthetic selective media + 2% dextrose at 30°C, and cultures were spotted on the indicated media in 10-fold serial dilutions using a multi-prong applicator (Dan-Kar), and grown at 30°C for two to three days.

Quantitative mating was performed as described previously [61]. Tested strains and tester strains were placed on YEP + 2% glucose plates and grown overnight at 30°C. On the following morning, the cells were scraped from the plates, and heavily inoculated into YEP + 2% dextrose liquid medium and grown for two to four hours at 25°C to an OD_600_ of one to two for the tested strains and to an OD_600_ of four to six for the tester strains. Quantities of 10^6^, 10^5^, 10^4^, 10^3^, and 10^2^ cells of the tested strain (in 100μl) were mixed with 10^7^ to 5 X 10^7^ cells of the tester strain (in 300μl). The mixture was plated directly on synthetic minimal + 2% dextrose plates and grown at 25°C for 3 days, and then colony counting was performed to determine the mating efficiency. A quantity of 10^2^ cells of the tested strain was also plated on synthetic complete + 2% dextrose plates to accurately determine the number of viable cells that were mated in the experiment. Within each experiment, matings were done in duplicate, and at least three independent mating experiments were performed. Mating efficiencies are typically between 50 and 100% for wild type cells (ADR21 and ADR22). The matings in Figs 2D, 5B and 5C and 6E were performed by different individuals (S.P., C.D. and A.D.R.) and accounts for the differences in efficiencies between identical (JRY8828 X JRY8829) and similar (ADR21 and ADR22) strains.

*hmrΔA*::*ADE2* cells were grown at 30°C overnight in liquid YEP + 2% dextrose media, 200–500 cells plated on YEP + 2% dextrose plates and grown for 3 days at 30°C. The plates were then left at 4 degrees to allow the red color to develop. Cells were scored for their ability to completely silence/express the *ADE2* locus (red or white) or switch between silenced and unsilenced states (sectored colonies).

### Western blots

These methods have been described previously [61,101]. Yeast extracts for Western blotting were made by bead beating (multitube bead beater; Biospec) frozen cell pellets in 1X sample buffer (2% SDS, 80mM Tris-HCl pH6.8, 10% glycerol, 10mM EDTA, bromophenol blue, 5% 2-mercaptoethanol). Samples were normalized by cell concentration before harvesting.

Standard methods were used for polyacrylamide gel electrophoresis and protein transfer to nitrocellulose (Pall). Typically, samples were run on 12.5% polyacrylamide gels (120:1 acrylamide:bis, respectively, with no added SDS). Blots were stained with Ponceau S to confirm transfer and equal loading of samples, and then were blocked for 30 min in blocking buffer (4% nonfat dried milk (Carnation) in TBST (20mM Tris-HCl (pH 7.5), 150mM NaCl, 0.1% Tween 20). All antibodies were incubated overnight at 4°C or for 2 h at 25°C. After washing with TBST, the blots were incubated with horseradish peroxidase-conjugated anti-rabbit antibodies (Bio-Rad) at a 1:5,000 dilution in blocking buffer for 30 min at 25°C, washed again, incubated with Western Lightning reagents (Perkin-Elmer) and then exposed to X-Omat film (Kodak) or imaged on a GE Image Quant LAS4010 (GE) imaging system. Densitometry of bands was performed using ImageQuant TL (GE) software.

Affinity-purified rabbit polyclonal anti-Sir2 [102] and anti-Sir4 antibodies were used at a dilution of 1:2,500, and anti-Sir3 and anti-histone H4 (Millipore; 04–858) were used at a dilution of 1:1000, in antibody storage buffer (autoclaved 4% nonfat dried milk, TBST, 5% glycerol, 0.02% NaN3). Anti-Cdk1 antibody [101] was used at a dilution of 1:1000 in BSA storage buffer (sterile 2% BSA, TBST, 0.02% NaN3).

Anti-Sir3 and anti-Sir4 antibodies were generated as follows. His6-Sir3 (aa522-978) and GST-Sir4 (aa1165-1358) was expressed in bacteria from pAR1007 and pAR411, respectively (kindly provided by Danesh Moazed, Harvard Medical School, Boston, MA). 1mg of each fusion protein was injected into rabbits every 4 weeks for 8 to 16 weeks (uOttawa animal facility). Rabbit serum was harvested, clarified by centrifugation and loaded on Affigel-10 (Bio-rad) columns coupled to purified His6-Sir3 (aa522-978) and malE-Sir4 (aa1165-1358), respectively. malE-Sir4 (aa1165-1358) was expressed from pAR653 in which Sir4 (aa1165-1358) was cloned as BamH1/Sal1 fragments into pMAL-c2 (NEB). Antibody was eluted from Affigel columns with either 100mM triethylamine pH 11.5 or 100mM glycine pH 2.3. The triethylamine and glycine elutions were neutralized, dialyzed in PBS + 50% glycerol and stored at -80°C.

### Chromatin fractionation

Chromatin fractionation was performed as described previously [103]. 0.5 X 10^9^ cells at ~2 X 10^7^ cells/ml were harvested and sodium azide was added to 0.1%. Cells were washed once in pre-spheroplasting buffer (100mM PIPES (pH 9.4), 10mM DTT) and then incubated for ten minutes at room temperature in 5 ml of the same buffer. Cells were collected by centrifugation and resuspended in 1 ml of spheroplasting buffer (50mM KH_2_PO_4_/ K_2_HPO_4_ (pH 7.5), 0.6M Sorbitol, 10mM DTT) containing 50μl of 2mg/ml Lyticase (Sigma) and incubated at room temperature with end-over-end mixing for 10–20 minutes. Spheroplasting was considered complete when the OD600 of a 1:100 dilution of the cell suspension (in water) dropped to <10% of the value before digestion. Spheroplasts were washed with 1 ml of ice-chilled wash buffer (100mM KCl, 50mM HEPES-KOH (pH 7.5), 2.5mM MgCl_2_, and 0.4M Sorbitol), pelleted at 4000 rpm for 1 min in a microcentrifuge at 4°C, and resuspended in 500μl of extraction buffer (100mM KCl, 50mM HEPES-KOH (pH 7.5), 2.5mM MgCl_2_, 50mM NaF, 1mM NaVO_3_, and added fresh: 1mM PMSF, 1mM benzamidine HCl, and 10μg/ml of leupeptin, pepstatin, bestatin and chymostatin). Spheroplasts were lysed by adding Triton X-100 to 0.25% and incubating on ice for 5 min with gentle mixing. A portion of the total lysate (T) was removed and mixed with 2X sample buffer, and the remaining lysate was spun at 12,000 rpm for 10 min at 4°C. The supernatant (S) was removed and a portion mixed with 2X sample buffer. The crude chromatin pellet (C) was washed with extraction buffer containing 0.25% Triton X-100, and spun again at 10,000 rpm for 5 min at 4°C. The pellet was resuspended in the wash buffer and then mixed with 2X sample buffer. Fractions were heated at 65°C for ten minutes and equal cell equivalent volumes were run on polyacrylamide gels (12.5% and 20%) and processed for western blotting with the indicated antibodies.

### Chromatin immunoprecipitation

Chromatin immunoprecipitation was performed as described previously {Rudner:2005fd}. Sir4 was precipitated with 1μl of affinity purified rabbit polyclonal anti-Sir4 antibody, histone H3 was precipitated with 1μl of rabbit anti-histone H3 antibody (Millipore; 04–928) and histone H3 methylated on K79 was precipitated with 1μl of rabbit anti histone H3-pan-methyl K79 antibody (AbCam; ab28940). PCR was performed with 0.1mCi [*α*-^32^P] dCTP (3,000 Ci/mmol; Perkin Elmer), reactions were resolved on 6% acrylamide (30:1 acrylamide:bis)-Tris-borate-EDTA gels, and quantified by phosphorimaging on a Typhoon Trio phosphorimager and ImageQuant software (GE Healthcare).

Relative fold enrichment values for each strain were calculated as follows: [silent locus (immunoprecipitate)/*ACT1* (immunoprecipitate)]/[silent locus (input)/*ACT1* (input)]. The average and standard error for three independent experiments were determined. For clarity, these values were scaled so that the average values of *sir4Δ* in the Sir4 ChIP was set to one; the average values were scaled so that the *dot1Δ* values in the histone H3 ChIP was set to one.

## Supporting Information

S1 Fig Sir4 protein is degraded during stationary phase and is maintained at existing heterochromatic loci in mating pheromone. *(A)* Asynchronously growing (asyn) wild type (ADR4006) cells were grown for 24 hours in YEP + 2% raffinose at 25°C and then diluted into fresh YEP + 2% dextrose in the absence or presence of nocodazole (N). Samples were harvested at the indicated times and protein levels were analyzed by western blot. Cdk1 is shown as a loading control. *(B)* Asynchronously (asyn) growing wild type (ADR4006) cells were arrested in G1 with 1μg/ml α-factor or arrested in mitosis with 10μg/ml nocodazole at 25°C for five hours. Cells were fixed in 1% formaldehyde for fifteen minutes and processed for ChIP with anti-Sir4 polyclonal antibodies. *sir4Δ* cells (ADR3387) were grown asynchronously. The localization Sir4 to the indicated loci was determined by analyzing the immunoprecipitated DNA by PCR with locus-specific primers. Every PCR also contained primers to amplify a non-silent locus, *ACT1*, as an internal control for the input DNA, the immunoprecipitation, and the PCR. The *y* axis is the fold enrichment of PCR products amplified from immunoprecipitated DNA relative to that of products from input DNA and is the average and SEM of three independent experiments. For clarity, the enrichment of the *sir4Δ* strain is arbitrarily set to 1. *(C) SIR4-eGFP* (ADR3810) cells were arrested in G1 with 1μg/ml α-factor or arrested in mitosis with 10μg/ml nocodazole at 25°C for five hours, fixed and imaged by fluorescence microscopy. Bright field (BF) and GFP fluorescence (Sir4-GFP) example cells are shown. *(D)* The intensity of Sir4-GFP foci (n = 24 for both conditions) were measured relative to background fluorescence under both treatments and compared. Although variance of the foci was greater in G1 (left panel), the average intensity (mean +/- SEM) was not statistically significant between G1 and mitosis (right panel) (p = 0.254, Student’s two-tailed t-test). Background fluorescence was determined in *SIR4-eGFP* and wild type cells (ADR4006) grown in both conditions (n = 12 for each) and the average background intensity was not statistically significant between the four conditions. *(E)* Wild type (ADR4006) cells were arrested in G1 with 1μg/ml α-factor (αf) or arrested in mitosis with 10μg/ml nocodazole (noc) at 25°C for five hours. One set of samples were lysed directly in sample buffer (crude lysate) and analyzed by western blot. Two-fold dilutions of the nocodazole sample were loaded to assist in quantification of samples. A second set of samples were lysed as described by Liang *et al*. ([103] and see Materials and Methods), and the total lysate (T) was separated into a soluble fraction (S) and a chromatin fraction (C) by centrifugation. Two-fold dilutions of the total lysate were loaded to assist in quantification of samples. Greater than 50% of the Sir4 and 75% of the Sir2 is present in the chromatin fraction. The slower migrating form of Sir4 that is only present in the soluble fraction was observed in several experiments. Cdk1 and Histone H4 serve as controls for the soluble and chromatin fraction respectively. A background band that reacts with the anti-histone H4 antibody (*) is predominantly soluble.
(TIF)

S2 Fig *SIR4* is haploinsufficient for subtelomeric silencing. *(A)* Strains containing *URA3* at *TELVII-L* or at *ADH4* [104] were mated to form *SIR4/SIR4 TELVII-L*::*URA3* (ADR2828 X ADR21), *SIR4/SIR4 adh4*::*URA3* (ADR21 X ADR2830) and *sir4Δ/SIR4 TELVII-L*::*URA3* (ADR2828 X ADR3344) diploids. To allow mating, *sir4Δ* cells contained a *SIR4-CEN-HIS3* plasmid (pAR450) which was lost before silencing was assayed. The ability to silence *URA3* transcription was measured by the ability of ten-fold serial dilutions of cells to grow on plates containing 5-FOA. *URA3* inserted at the internal *ADH4* locus is not silenced. Papillation of strains that do not grow on SC+FOA is likely caused by loss of *TELVII-L* and *URA3*. Our results suggest that this silencing defect is caused by a combination of an establishment defect and the inherent instability of subtelomeric heterochromatin. *(B)* Haploid cells that were either *SIR4* or *sir4*Δ were mated to form *SIR4/sir4Δ* (JRY8828 X ADR4593) and *sir4Δ/SIR4* (ADR4592 X JRY8829) diploid zygotes which were monitored for establishment of silencing at *HMLα* and categorized as in Fig 2A. There is no statistical significance between the two different *sir4Δ/SIR4* heterozygotes, or the combined data.
(TIF)

S3 Fig Additional *SIR4* improves silencing, increases Sir4 and speeds *de novo* establishment. *(A)* Wild type (ADR4062) and *sir4Δ* (ADR4482) cells containing *TELVII-L*::*URA3 hmrΔE*::*TRP1* and a *SIR4-CEN-HIS3* plasmid (pAR646) or an empty *CEN-HIS3* plasmid (pRS313) were grown for two days in SC-HIS liquid media at 30°C, and ten-fold serial dilutions were spotted on SC-HIS, SC-HIS+FOA and SC-HIS-TRP plates and grown for two to three days before photographing. Additional *SIR4* improves silencing as reported by Sussel *et al*. [35]. *(B) SIR4-CEN-HIS3* and *SIR4-CEN-URA3* plasmids (pAR646 and pAR722), or empty *CEN-HIS3* and *CEN-URA3* (pRS313 and pRS316) were transformed into the two *SIR4* mating strains (JRY8828, left panel and JRY8829, right panel). Cells were grown overnight under selection, harvested and protein levels were analyzed by western blot. Cdk1 serves as a loading control and *sir4Δ* cells (ADR3387) were used as a control for blotting. Two-fold serial dilutions of the 2 *SIR4-CEN* samples were analyzed to assess Sir4 concentration. *(C) SIR4* cells (JRY8828 and JRY8829) with or without empty centromeric plasmids (pRS313 and pRS316) were mated to form diploid zygotes which were monitored for establishment of silencing at *HMLα* and categorized as in Fig 2A. There is no statistical difference between the profiles of the three strains. *(D) SIR4* transcription varies with induction by β-estradiol. Cells containing *pGAL-SIR4* (ADR5389) and a hormone inducible Gal4-ER-VP16 (GEV, pAR917) [38] were grown in the indicated concentrations of β-estradiol in liquid culture at 25°C and Sir4 protein expression was analyzed by western blot. Cdk1 serves as a loading control. *(E) pGAL-SIR4* cells with the integrated β-estradiol construct (ADR5389 X ADR5390) were grown overnight at 30°C on YEP + 2% raffinose plates prior to mating on YEP + 2% raffinose plates containing no drug or 350nM β-estradiol, or YEP + 2% dextrose plates. Zygotes were monitored for establishment of silencing at *HMLα* and categorized as in Fig 2A.
(TIF)

S4 Fig Overexpression of Sir4 disrupts silencing and slows or prevents *de novo* establishment. *(A)* Wild type cells (JRY8828 X JRY8829) containing *SIR4-2μ-HIS3* plasmid (pAR696) or an empty *2μ-HIS3* plasmid (pAR423) were mated and pedigrees from the resulting zygotes were monitored for establishment of silencing at *HMLα* and categorized as described in Fig 2A. *(B)* Wild type (ADR4006) or *sir4Δ* (ADR3387) containing the *SIR4-2μ-HIS3* plasmid (pAR696) or a *CEN-HIS3* (pRS313) plasmid were grown in selective media, harvested, and protein levels were analyzed by western blot. Two-fold serial dilutions of the *sir4Δ + SIR4-2μ* sample was used to estimate the increase of expression of Sir4. Sir2 serves as a loading control. *(C)* Wild type (ADR4062) and *sir4Δ* (ADR4482) cells containing *TELVII-L*::*URA3 hmrΔE*::*TRP1* and a *SIR4-2μ-HIS3* plasmid (pAR696) or an empty *2μ-HIS3* plasmid (pRS423) were grown for two days in SC-URA liquid media at 30°C, and ten-fold serial dilutions were spotted on SC-HIS, SC-HIS+FOA and SC-HIS-TRP plates and grown for two to three days before photographing. Overexpression of *SIR4* disrupts silencing as reported previously [39]. *(D) SIR4* (JRY8828 X JRY8829) or *pGAL-SIR4* (ADR4562 X ADR4564) cells were mated and pedigrees from the resulting zygotes categorized as described in Fig 2A. Growth prior, during and after mating was done on YEP + 2% galactose. *(E) SIR4* (JRY8828) or *pGAL-SIR4* (ADR4562) were grown in YEP + 2% galactose liquid media and samples were harvested and analyzed by western blot. Two-fold serial dilutions of the *pGAL-SIR4* sample was used to estimate the increase of expression of Sir4. Cdk1 levels are shown as a loading control.
(TIF)

S5 Fig Deletion of *YKU70*, *DOT1* and *UBP10* disrupt silencing of reporters at telomeres and *HMR*, and are suppressed by deletion of *RIF1* and *RIF2*. ***(A)*** Wild type (ADR4062), *sir3Δ* (ADR5469), *yku70Δ* (ADR5840), *dot1Δ* (ADR5895) and *ubp10Δ* (ADR5843) cells containing *TELVII-L*::*URA3 hmrΔE*::*TRP1* were grown for two days in YEP + 2% dextrose liquid media at 30°C, and ten-fold serial dilutions were spotted on SC, SC+FOA and SC-TRP plates, and grown for two to three days before photographing. *(B)* Wild type (ADR4062), *rif1Δ* (ADR8969), *rif2Δ* (ADR8953), *rif1Δ rif2Δ* (ADR8974), *yku70Δ* (ADR5840), *yku70Δ rif1Δ* (ADR8901), *yku70Δ rif2Δ* (ADR8903), *yku70Δ rif1Δ rif2Δ* (ADR8907), *dot1Δ* (ADR5895), *dot1Δ rif1Δ* (ADR8936), *dot1Δ rif2Δ* (ADR8939), *dot1Δ rif1Δ rif2Δ* (ADR8944), *ubp10Δ* (ADR5843), *ubp10Δ rif1Δ* (ADR8958), *ubp10Δ rif2Δ* (ADR8961) and *ubp10Δ rif1Δ rif2Δ* (ADR8962) cells containing *TELVII-L*::*URA3 hmrΔE*::*TRP1* were grown for two days in YEP + 2% dextrose liquid media at 30°C, and ten-fold serial dilutions were spotted on SC, SC+FOA and SC-TRP plates, and grown for two to three days before photographing. *(C)* Wild type (GCY317), *esc8Δ* (GCY310), *dot1Δ* (ADR6184), *ubp10Δ* (ADR6185) and *yku70Δ* (ADR6186) cells containing the *hmrΔA*::*ADE2* reporter were grown at 30°C overnight in liquid YEP + 2% dextrose media, 200–500 cells plated on YEP + 2% dextrose plates and grown for 3 days at 30°C. The plates were then left at 4 degrees to allow the red color to develop. Cells were scored for their ability to completely silence or express the *ADE2* locus (red or white) or switch between silenced and unsilenced states (sectored colonies). Data graphed is the mean of at least three independent experiments per strain (ADR6144 n = 854, ADR6145 n = 1111, ADR6184 n = 452, ADR6185 n = 203, ADR6186 n = 396).
(TIF)

S6 Fig Deletion of *YKU70* and *UBP10* does not alter histone H3 K79 methylation at *HMLα*. Asynchronously growing wild type (ADR22), *dot1Δ* (ADR6181), *ubp10Δ* (ADR6182) and *yku70Δ* (ADR6183) cells were fixed in 1% formaldehyde for fifteen minutes and processed for ChIP with anti-Sir4 polyclonal antibodies. The relative enrichment of Sir4 and pan methylation on K79 of histone H3 (pMeK79-H3) at the indicated loci was determined by analyzing the immunoprecipitated DNA by PCR with locus-specific primers. Every PCR also contained primers to amplify a non-silent locus, *ACT1*, as an internal control for the input DNA, the immunoprecipitation, and the PCR. The pMeK79-H3 ChIP was normalized relative to the enrichment of total histone H3. The *y* axis is the fold enrichment of PCR products amplified from immunoprecipitated DNA relative to that of products from input DNA and is the average and SEM of three independent experiments. For clarity, the enrichment of the *sir4*Δ and *dot1Δ* strain are arbitrarily set to 1 in the Sir4 ChIP and pMeK79-H3 ChIP, respectively.
(TIF)

S7 Fig Additional single cell establishment assays. *(A)* Cells were mated to create *SIR4/SIR4* (JRY8828 X JRY8829), *sir4Δ/SIR4* (ADR4592 X JRY8829 and JRY8828 X ADR4593), *ubp10Δ/ubp10Δ* (ADR5087 X ADR5088), and *sir4Δ ubp10Δ/SIR4 ubp10Δ* (ADR5550 X ADR5088 or ADR5087 X ADR5551), and the resulting zygotes were monitored and categorized as in Fig 2A. *(B)* Cells were mated to create *SIR4/SIR4* (JRY8828 X JRY8829), *sir4Δ/SIR4* (ADR4592 X JRY8829 and JRY8828 X ADR4593), *yku70Δ/yku70Δ* (ADR5841 X ADR5842), and *sir4Δ yku70Δ/SIR4 yku70Δ* (ADR7842 X ADR5842 or ADR5841 X ADR7846), and the resulting zygotes were monitored and categorized as in Fig 2A. *(C)* Haploid *SIR4* (JRY8828 and JRY8829), *dot1Δ* (ADR4631 and ADR4632), *ubp10Δ* (ADR5087 and ADR5088) and *dot1Δ ubp10Δ* (ADR5171 and ADR5172) cells were mated to produce homozygous zygotes that were monitored for establishment of silencing at *HMLα* and categorized as described in Fig 2A. *(D)* Haploid *SIR4* (JRY8828 and JRY8829), *dot1Δ* (ADR4631 and ADR4632), *yku70Δ* (ADR5841 and ADR5842) and *dot1Δ yku70Δ* (ADR5944 and ADR5945) cells were mated to produce homozygous zygotes that were monitored for establishment of silencing at *HMLα* and categorized as described in Fig 2A. *(E)* Haploid *SIR4* (JRY8828 and JRY8829), *ubp10Δ* (ADR5087 and ADR5088), *yku70Δ* (ADR5841 and ADR5842) and *ubp10Δ yku70Δ* (ADR5920 and ADR5921) cells were mated to produce homozygous zygotes that were monitored for establishment of silencing at *HMLα* and categorized as described in Fig 2A. *(F)* Haploid *SIR4* (JRY8828 and JRY8829), *rif1Δ* (ADR7962 and ADR7966), *yku70Δ rif1Δ* (ADR7972 X ADR7975) and *yku70Δ rif1Δ rif2Δ* (ADR7986 and ADR7989) cells were mated to produce homozygous zygotes that were monitored for establishment of silencing at *HMLα* and categorized as described in Fig 2A. The distribution of the four strains are not significantly different. Statistics for every pairwise comparison can be found in S1 Table.
(TIF)

S1 Table Statistics for every pairwise comparison for all pedigree assays. (DOCX)

S2 Table Complete list of all yeast strains used in this study. (DOCX)
