# Supplementary figures and images for: Competition between Heterochromatic Loci Allows the Abundance of the Silencing Protein, Sir4, to Regulate de novo Assembly of Heterochromatin
Source: PLoS Genet. 2015 Nov 20;11(11):e1005425. doi: 10.1371/journal.pgen.1005425 (PMC4654584; doi:10.1371/journal.pgen.1005425)

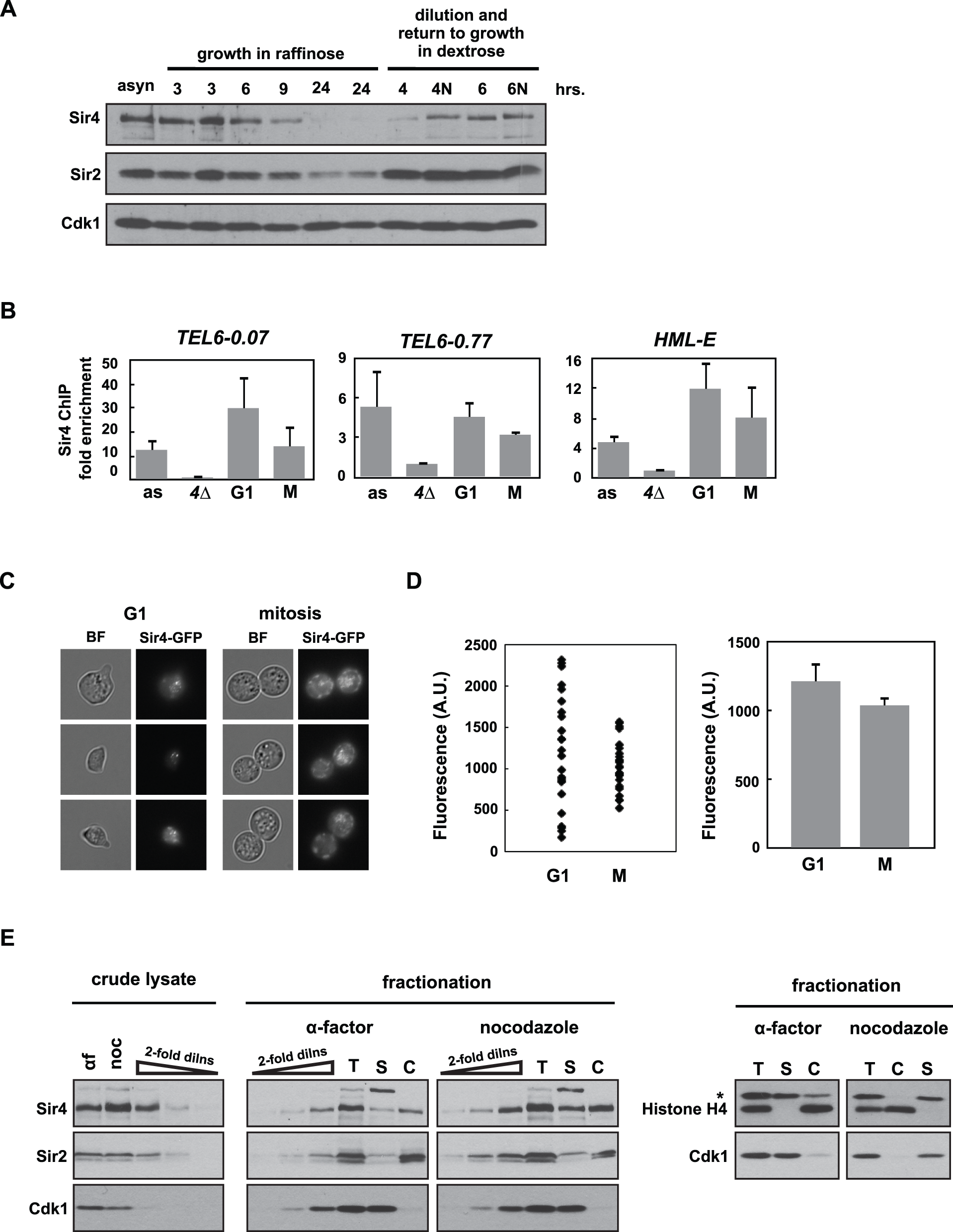

Supplement: S1 Fig — (A) Asynchronously growing (asyn) wild type (ADR4006) cells were grown for 24 hours in YEP + 2% raffinose at 25°C and then diluted into fresh YEP + 2% dextrose in the absence or presence of nocodazole (N). Samples were harvested at the indicated times and protein levels were analyzed by western blot. Cdk1 is shown as a loading control. (B) Asynchronously (asyn) growing wild type (ADR4006) cells were arrested in G1 with 1μg/ml α-factor or arrested in mitosis with 10μg/ml nocodazole at 25°C for five hours. Cells were fixed in 1% formaldehyde for fifteen minutes and processed for ChIP with anti-Sir4 polyclonal antibodies. sir4Δ cells (ADR3387) were grown asynchronously. The localization Sir4 to the indicated loci was determined by analyzing the immunoprecipitated DNA by PCR with locus-specific primers. Every PCR also contained primers to amplify a non-silent locus, ACT1, as an internal control for the input DNA, the immunoprecipitation, and the PCR. The y axis is the fold enrichment of PCR products amplified from immunoprecipitated DNA relative to that of products from input DNA and is the average and SEM of three independent experiments. For clarity, the enrichment of the sir4Δ strain is arbitrarily set to 1. (C) SIR4-eGFP (ADR3810) cells were arrested in G1 with 1μg/ml α-factor or arrested in mitosis with 10μg/ml nocodazole at 25°C for five hours, fixed and imaged by fluorescence microscopy. Bright field (BF) and GFP fluorescence (Sir4-GFP) example cells are shown. (D) The intensity of Sir4-GFP foci (n = 24 for both conditions) were measured relative to background fluorescence under both treatments and compared. Although variance of the foci was greater in G1 (left panel), the average intensity (mean +/- SEM) was not statistically significant between G1 and mitosis (right panel) (p = 0.254, Student’s two-tailed t-test). Background fluorescence was determined in SIR4-eGFP and wild type cells (ADR4006) grown in both conditions (n = 12 for each) and the average backgrou [file pgen.1005425.s001.tif]

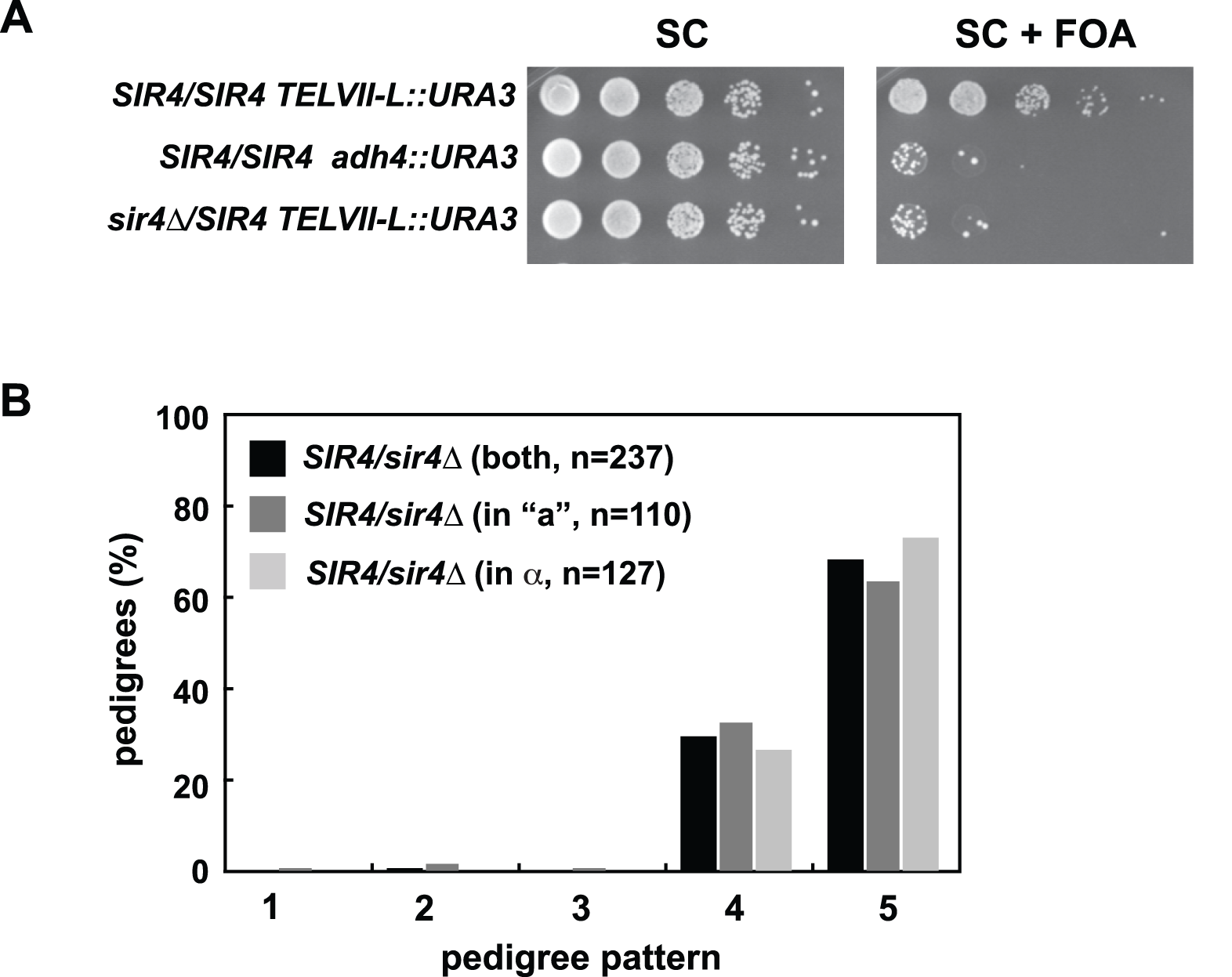

Supplement: S2 Fig — (A) Strains containing URA3 at TELVII-L or at ADH4 [104] were mated to form SIR4/SIR4 TELVII-L::URA3 (ADR2828 X ADR21), SIR4/SIR4 adh4::URA3 (ADR21 X ADR2830) and sir4Δ/SIR4 TELVII-L::URA3 (ADR2828 X ADR3344) diploids. To allow mating, sir4Δ cells contained a SIR4-CEN-HIS3 plasmid (pAR450) which was lost before silencing was assayed. The ability to silence URA3 transcription was measured by the ability of ten-fold serial dilutions of cells to grow on plates containing 5-FOA. URA3 inserted at the internal ADH4 locus is not silenced. Papillation of strains that do not grow on SC+FOA is likely caused by loss of TELVII-L and URA3. Our results suggest that this silencing defect is caused by a combination of an establishment defect and the inherent instability of subtelomeric heterochromatin. (B) Haploid cells that were either SIR4 or sir4Δ were mated to form SIR4/sir4Δ (JRY8828 X ADR4593) and sir4Δ/SIR4 (ADR4592 X JRY8829) diploid zygotes which were monitored for establishment of silencing at HMLα and categorized as in Fig 2A. There is no statistical significance between the two different sir4Δ/SIR4 heterozygotes, or the combined data. (TIF) [file pgen.1005425.s002.tif]

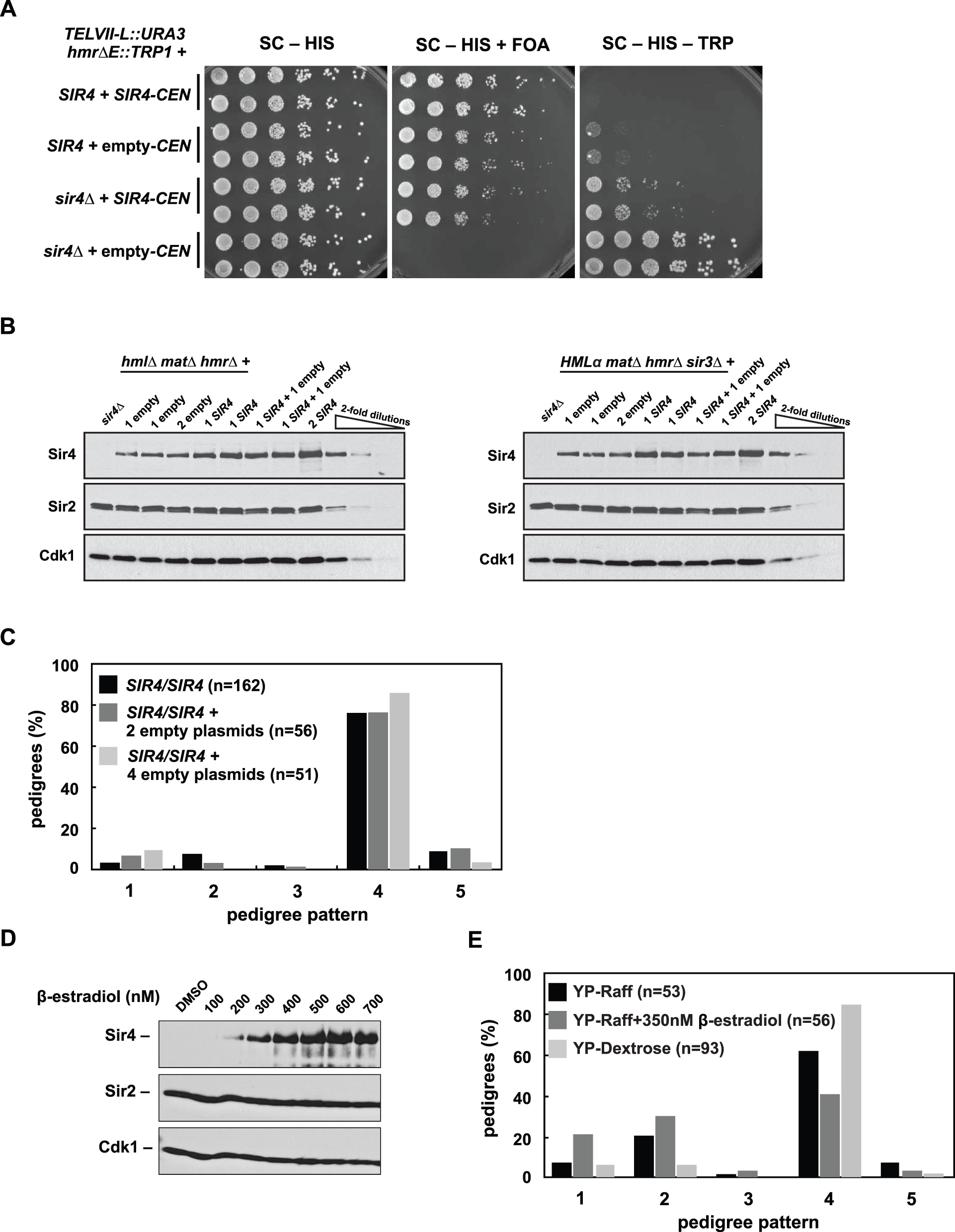

Supplement: S3 Fig — (A) Wild type (ADR4062) and sir4Δ (ADR4482) cells containing TELVII-L::URA3 hmrΔE::TRP1 and a SIR4-CEN-HIS3 plasmid (pAR646) or an empty CEN-HIS3 plasmid (pRS313) were grown for two days in SC-HIS liquid media at 30°C, and ten-fold serial dilutions were spotted on SC-HIS, SC-HIS+FOA and SC-HIS-TRP plates and grown for two to three days before photographing. Additional SIR4 improves silencing as reported by Sussel et al. [35]. (B) SIR4-CEN-HIS3 and SIR4-CEN-URA3 plasmids (pAR646 and pAR722), or empty CEN-HIS3 and CEN-URA3 (pRS313 and pRS316) were transformed into the two SIR4 mating strains (JRY8828, left panel and JRY8829, right panel). Cells were grown overnight under selection, harvested and protein levels were analyzed by western blot. Cdk1 serves as a loading control and sir4Δ cells (ADR3387) were used as a control for blotting. Two-fold serial dilutions of the 2 SIR4-CEN samples were analyzed to assess Sir4 concentration. (C) SIR4 cells (JRY8828 and JRY8829) with or without empty centromeric plasmids (pRS313 and pRS316) were mated to form diploid zygotes which were monitored for establishment of silencing at HMLα and categorized as in Fig 2A. There is no statistical difference between the profiles of the three strains. (D) SIR4 transcription varies with induction by β-estradiol. Cells containing pGAL-SIR4 (ADR5389) and a hormone inducible Gal4-ER-VP16 (GEV, pAR917) [38] were grown in the indicated concentrations of β-estradiol in liquid culture at 25°C and Sir4 protein expression was analyzed by western blot. Cdk1 serves as a loading control. (E) pGAL-SIR4 cells with the integrated β-estradiol construct (ADR5389 X ADR5390) were grown overnight at 30°C on YEP + 2% raffinose plates prior to mating on YEP + 2% raffinose plates containing no drug or 350nM β-estradiol, or YEP + 2% dextrose plates. Zygotes were monitored for establishment of silencing at HMLα and categorized as in Fig 2A. (TIF) [file pgen.1005425.s003.tif]

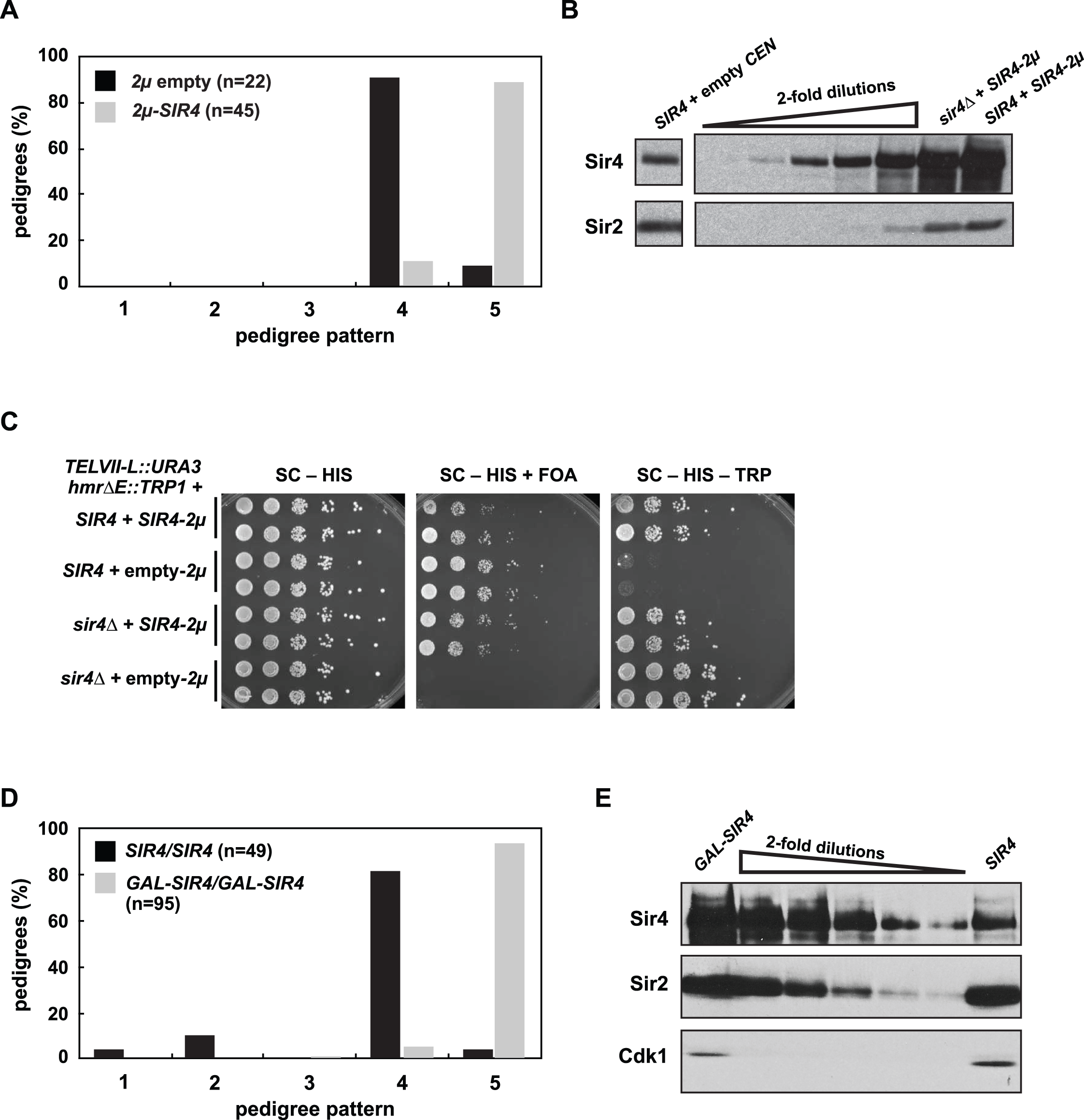

Supplement: S4 Fig — (A) Wild type cells (JRY8828 X JRY8829) containing SIR4-2μ-HIS3 plasmid (pAR696) or an empty 2μ-HIS3 plasmid (pAR423) were mated and pedigrees from the resulting zygotes were monitored for establishment of silencing at HMLα and categorized as described in Fig 2A. (B) Wild type (ADR4006) or sir4Δ (ADR3387) containing the SIR4-2μ-HIS3 plasmid (pAR696) or a CEN-HIS3 (pRS313) plasmid were grown in selective media, harvested, and protein levels were analyzed by western blot. Two-fold serial dilutions of the sir4Δ + SIR4-2μ sample was used to estimate the increase of expression of Sir4. Sir2 serves as a loading control. (C) Wild type (ADR4062) and sir4Δ (ADR4482) cells containing TELVII-L::URA3 hmrΔE::TRP1 and a SIR4-2μ-HIS3 plasmid (pAR696) or an empty 2μ-HIS3 plasmid (pRS423) were grown for two days in SC-URA liquid media at 30°C, and ten-fold serial dilutions were spotted on SC-HIS, SC-HIS+FOA and SC-HIS-TRP plates and grown for two to three days before photographing. Overexpression of SIR4 disrupts silencing as reported previously [39]. (D) SIR4 (JRY8828 X JRY8829) or pGAL-SIR4 (ADR4562 X ADR4564) cells were mated and pedigrees from the resulting zygotes categorized as described in Fig 2A. Growth prior, during and after mating was done on YEP + 2% galactose. (E) SIR4 (JRY8828) or pGAL-SIR4 (ADR4562) were grown in YEP + 2% galactose liquid media and samples were harvested and analyzed by western blot. Two-fold serial dilutions of the pGAL-SIR4 sample was used to estimate the increase of expression of Sir4. Cdk1 levels are shown as a loading control. (TIF) [file pgen.1005425.s004.tif]

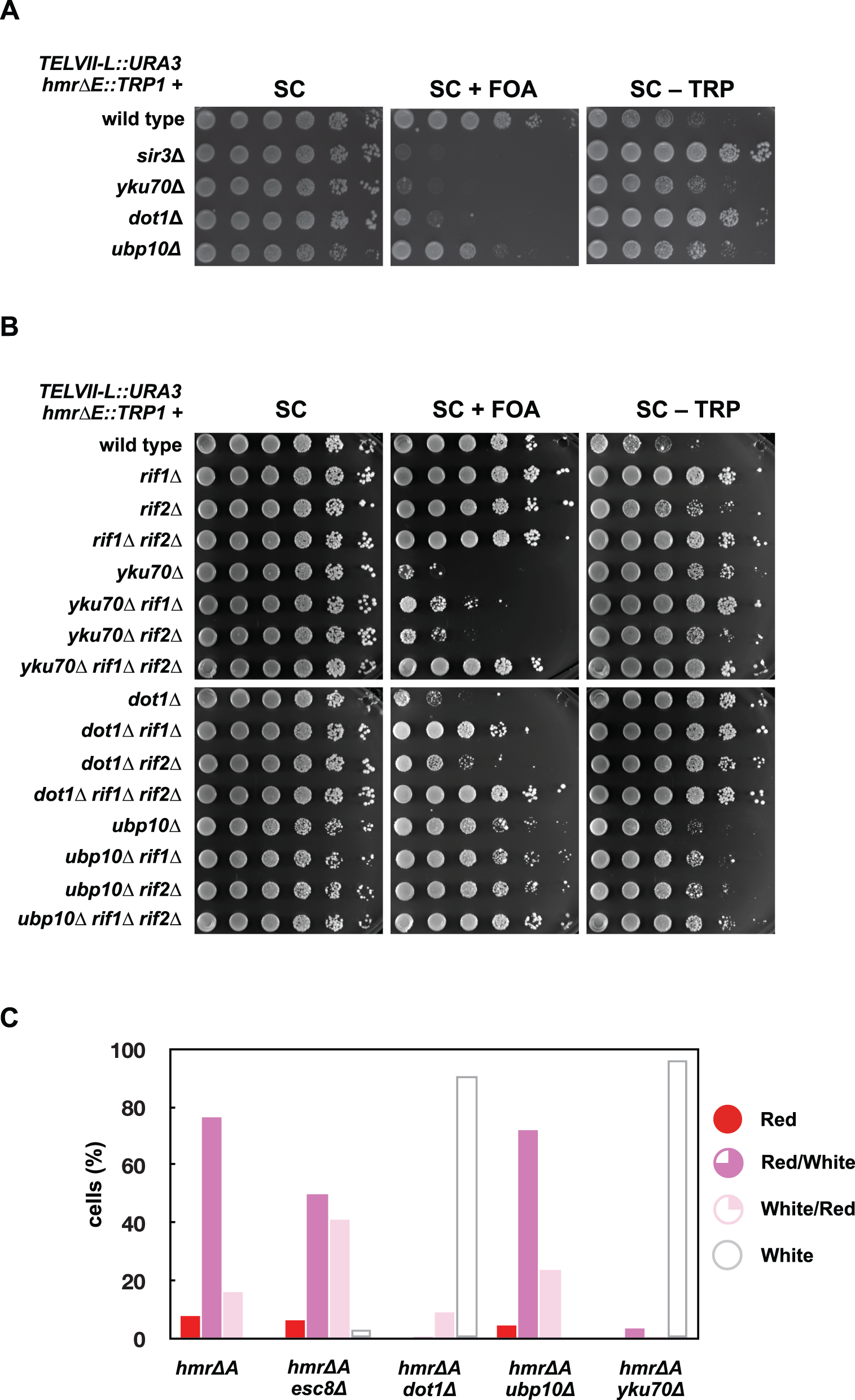

Supplement: S5 Fig — (A) Wild type (ADR4062), sir3Δ (ADR5469), yku70Δ (ADR5840), dot1Δ (ADR5895) and ubp10Δ (ADR5843) cells containing TELVII-L::URA3 hmrΔE::TRP1 were grown for two days in YEP + 2% dextrose liquid media at 30°C, and ten-fold serial dilutions were spotted on SC, SC+FOA and SC-TRP plates, and grown for two to three days before photographing. (B) Wild type (ADR4062), rif1Δ (ADR8969), rif2Δ (ADR8953), rif1Δ rif2Δ (ADR8974), yku70Δ (ADR5840), yku70Δ rif1Δ (ADR8901), yku70Δ rif2Δ (ADR8903), yku70Δ rif1Δ rif2Δ (ADR8907), dot1Δ (ADR5895), dot1Δ rif1Δ (ADR8936), dot1Δ rif2Δ (ADR8939), dot1Δ rif1Δ rif2Δ (ADR8944), ubp10Δ (ADR5843), ubp10Δ rif1Δ (ADR8958), ubp10Δ rif2Δ (ADR8961) and ubp10Δ rif1Δ rif2Δ (ADR8962) cells containing TELVII-L::URA3 hmrΔE::TRP1 were grown for two days in YEP + 2% dextrose liquid media at 30°C, and ten-fold serial dilutions were spotted on SC, SC+FOA and SC-TRP plates, and grown for two to three days before photographing. (C) Wild type (GCY317), esc8Δ (GCY310), dot1Δ (ADR6184), ubp10Δ (ADR6185) and yku70Δ (ADR6186) cells containing the hmrΔA::ADE2 reporter were grown at 30°C overnight in liquid YEP + 2% dextrose media, 200–500 cells plated on YEP + 2% dextrose plates and grown for 3 days at 30°C. The plates were then left at 4 degrees to allow the red color to develop. Cells were scored for their ability to completely silence or express the ADE2 locus (red or white) or switch between silenced and unsilenced states (sectored colonies). Data graphed is the mean of at least three independent experiments per strain (ADR6144 n = 854, ADR6145 n = 1111, ADR6184 n = 452, ADR6185 n = 203, ADR6186 n = 396). (TIF) [file pgen.1005425.s005.tif]

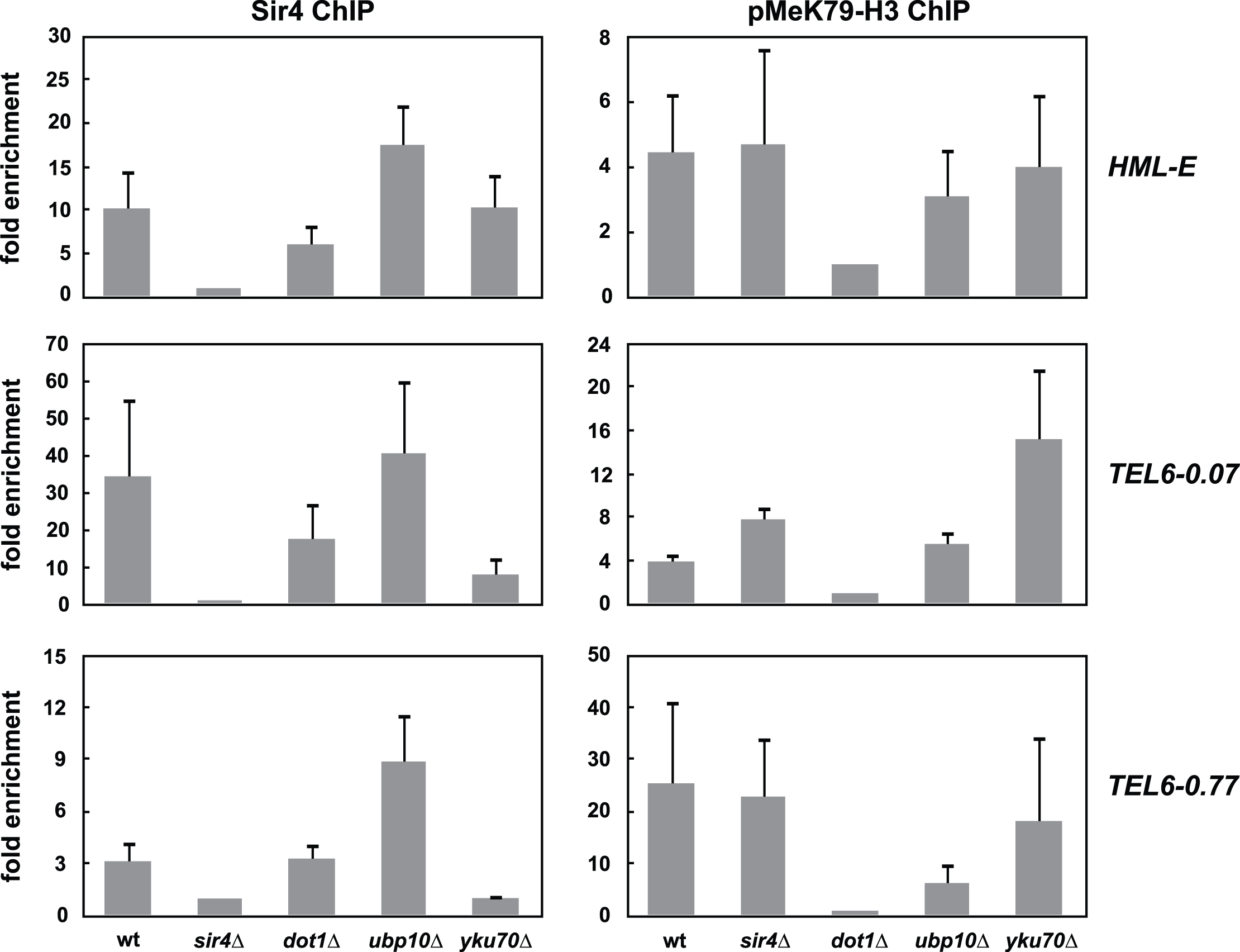

Supplement: S6 Fig — Asynchronously growing wild type (ADR22), dot1Δ (ADR6181), ubp10Δ (ADR6182) and yku70Δ (ADR6183) cells were fixed in 1% formaldehyde for fifteen minutes and processed for ChIP with anti-Sir4 polyclonal antibodies. The relative enrichment of Sir4 and pan methylation on K79 of histone H3 (pMeK79-H3) at the indicated loci was determined by analyzing the immunoprecipitated DNA by PCR with locus-specific primers. Every PCR also contained primers to amplify a non-silent locus, ACT1, as an internal control for the input DNA, the immunoprecipitation, and the PCR. The pMeK79-H3 ChIP was normalized relative to the enrichment of total histone H3. The y axis is the fold enrichment of PCR products amplified from immunoprecipitated DNA relative to that of products from input DNA and is the average and SEM of three independent experiments. For clarity, the enrichment of the sir4Δ and dot1Δ strain are arbitrarily set to 1 in the Sir4 ChIP and pMeK79-H3 ChIP, respectively. (TIF) [file pgen.1005425.s006.tif]

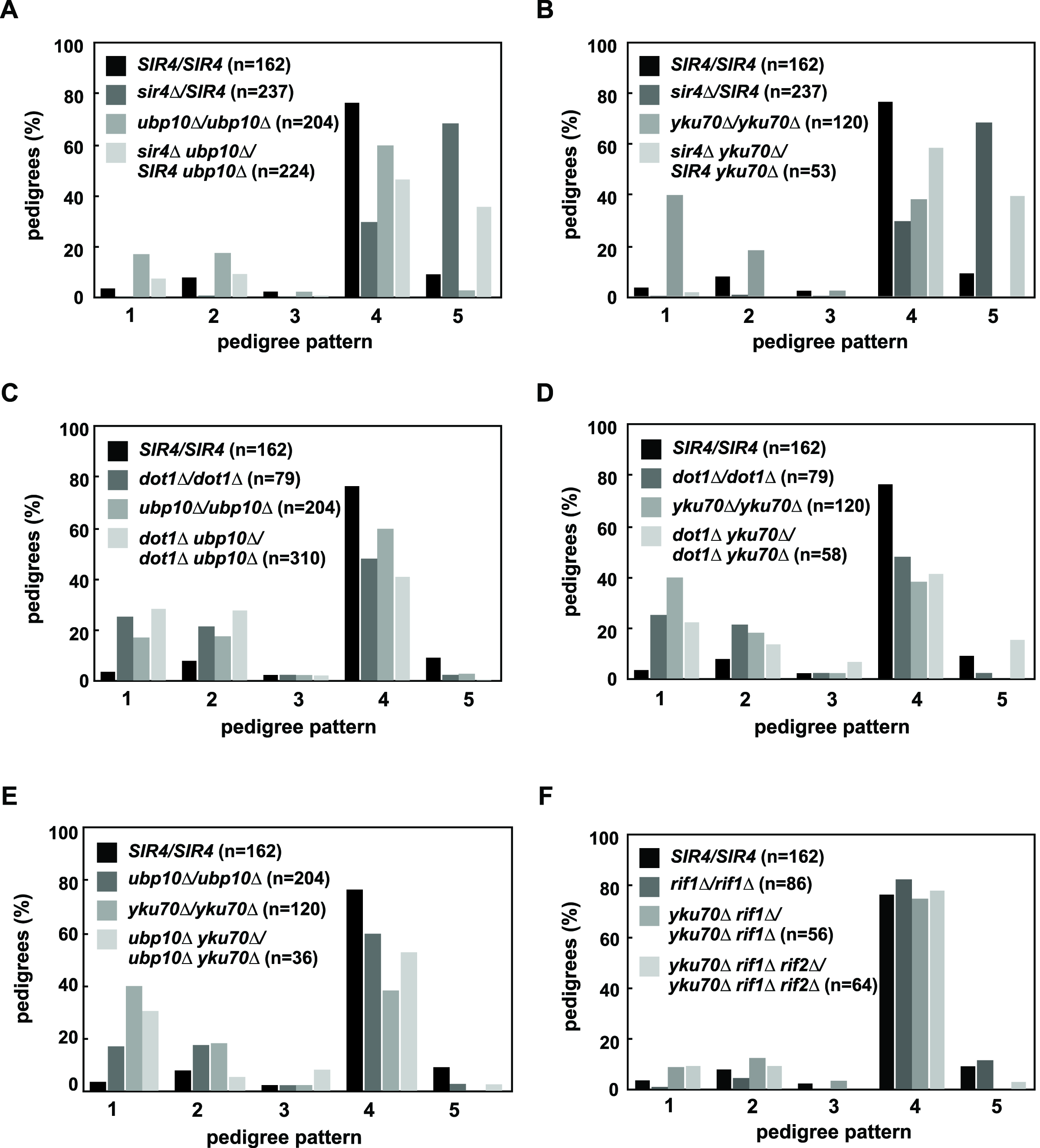

Supplement: S7 Fig — (A) Cells were mated to create SIR4/SIR4 (JRY8828 X JRY8829), sir4Δ/SIR4 (ADR4592 X JRY8829 and JRY8828 X ADR4593), ubp10Δ/ubp10Δ (ADR5087 X ADR5088), and sir4Δ ubp10Δ/SIR4 ubp10Δ (ADR5550 X ADR5088 or ADR5087 X ADR5551), and the resulting zygotes were monitored and categorized as in Fig 2A. (B) Cells were mated to create SIR4/SIR4 (JRY8828 X JRY8829), sir4Δ/SIR4 (ADR4592 X JRY8829 and JRY8828 X ADR4593), yku70Δ/yku70Δ (ADR5841 X ADR5842), and sir4Δ yku70Δ/SIR4 yku70Δ (ADR7842 X ADR5842 or ADR5841 X ADR7846), and the resulting zygotes were monitored and categorized as in Fig 2A. (C) Haploid SIR4 (JRY8828 and JRY8829), dot1Δ (ADR4631 and ADR4632), ubp10Δ (ADR5087 and ADR5088) and dot1Δ ubp10Δ (ADR5171 and ADR5172) cells were mated to produce homozygous zygotes that were monitored for establishment of silencing at HMLα and categorized as described in Fig 2A. (D) Haploid SIR4 (JRY8828 and JRY8829), dot1Δ (ADR4631 and ADR4632), yku70Δ (ADR5841 and ADR5842) and dot1Δ yku70Δ (ADR5944 and ADR5945) cells were mated to produce homozygous zygotes that were monitored for establishment of silencing at HMLα and categorized as described in Fig 2A. (E) Haploid SIR4 (JRY8828 and JRY8829), ubp10Δ (ADR5087 and ADR5088), yku70Δ (ADR5841 and ADR5842) and ubp10Δ yku70Δ (ADR5920 and ADR5921) cells were mated to produce homozygous zygotes that were monitored for establishment of silencing at HMLα and categorized as described in Fig 2A. (F) Haploid SIR4 (JRY8828 and JRY8829), rif1Δ (ADR7962 and ADR7966), yku70Δ rif1Δ (ADR7972 X ADR7975) and yku70Δ rif1Δ rif2Δ (ADR7986 and ADR7989) cells were mated to produce homozygous zygotes that were monitored for establishment of silencing at HMLα and categorized as described in Fig 2A. The distribution of the four strains are not significantly different. Statistics for every pairwise comparison can be found in S1 Table. (TIF) [file pgen.1005425.s007.tif]
