# Supplementary material for: Competition between Heterochromatic Loci Allows the Abundance of the Silencing Protein, Sir4, to Regulate de novo Assembly of Heterochromatin
Source: PLoS Genet. 2015 Nov 20;11(11):e1005425. doi: 10.1371/journal.pgen.1005425 (PMC4654584; doi:10.1371/journal.pgen.1005425)
Supplement: S1 Table — (DOCX) [file pgen.1005425.s008.docx]

|  | Control | Treatment | deviance* | p value |
| --- | --- | --- | --- | --- |
|  |  |  |  |  |
| Figure 2B | SIR4/SIR4 | SIR4/sir4∆ | 158.80 | <0.0000001 |
|  |  |  |  |  |
| Figure 3 | 1 CEN | 2 CEN | 13.40 | 0.0094 |
|  | 1 CEN | 4 CEN | 17.70 | 0.0014 |
|  | 2 CEN | 4 CEN | 5.50 | 0.24 |
|  | empty CEN | 1 CEN | 0.73 | 0.95 |
|  | empty CEN | 2 CEN | 16.93 | 0.0020 |
|  | empty CEN | 4 CEN | 20.75 | 0.00036 |
|  |  |  |  |  |
| Figure 4B | SIR4/SIR4 | dot∆/dot1∆ | 39.32 | <0.0000001 |
|  | SIR4/SIR4 | SIR4/sir4∆ | 158.80 | <0.0000001 |
|  | SIR4/SIR4 | dot1∆ sir4∆/dot1∆ SIR4 | 38.61 | <0.0000001 |
|  | dot∆/dot1∆ | dot1∆ sir4∆/dot1∆ SIR4 | 51.90 | <0.0000001 |
|  | dot∆/dot1∆ | SIR4/sir4∆ | 169.03 | <0.0000001 |
|  | SIR4/sir4∆ | dot1∆ sir4∆/dot1∆ SIR4 | 81.27 | <0.0000001 |
|  |  |  |  |  |
| Figure 5A | SIR4/SIR4 | dot∆/dot1∆ | 39.32 | <0.0000001 |
|  | SIR4/SIR4 | ubp10∆/ubp10∆ | 33.21 | 0.0000011 |
|  | SIR4/SIR4 | yku70∆/yku70∆ | 91.02 | <0.0000001 |
|  | dot∆/dot1∆ | ubp10∆/ubp10∆ | 3.80 | 0.44 |
|  | dot∆/dot1∆ | yku70∆/yku70∆ | 5.75 | 0.22 |
|  | ubp10∆/ubp10∆ | yku70∆/yku70∆ | 3.24 | 0.52 |
|  |  |  |  |  |
| Figure 6A | SIR4/SIR4 | yku70∆/yku70∆ | 91.02 | <0.0000001 |
|  | SIR4/SIR4 | ku70∆ rif1∆ rif2∆/ku70∆ rif1∆ rif2∆ | 7.99 | 0.092 |
|  | yku70∆/yku70∆ | ku70∆ rif1∆ rif2∆/ku70∆ rif1∆ rif2∆ | 37.22 | <0.0000001 |
|  |  |  |  |  |
| Figure 6B | SIR4/SIR4 | ubp10∆/ubp10∆ | 33.21 | 0.0000011 |
|  | SIR4/SIR4 | ubp10∆ rif1∆ rif2∆/ubp10∆ rif1∆ rif2∆ | 17.14 | 0.0018 |
|  | ubp10∆/ubp10∆ | ubp10∆ rif1∆ rif2∆/ubp10∆ rif1∆ rif2∆ | 24.77 | 0.00006 |
|  |  |  |  |  |
| Figure 6C | SIR4/SIR4 | dot1∆/dot1∆ | 39.32 | <0.0000001 |
|  | SIR4/SIR4 | dot1∆ rif1∆ rif2∆/dot1∆ rif1∆ rif2∆ | 59.38 | <0.0000001 |
|  | dot1∆/dot1∆ | dot1∆ rif1∆ rif2∆/dot1∆ rif1∆ rif2∆ | 12.26 | 0.015 |
|  |  |  |  |  |
| Figure 6D | SIR4/SIR4 | rif1∆/rif1∆ | 6.45 | 0.17 |
|  | SIR4/SIR4 | rif1∆ rif2∆/rif1∆ rif2∆ | 52.27 | <0.0000001 |
|  | rif1∆/rif1∆ | rif1∆ rif2∆/rif1∆ rif2∆ | 40.42 | <0.0000001 |
|  |  |  |  |  |
| Figure S2B | SIR4/sir4∆ in a | SIR4/sir4∆ in alpha | 7.64 | 0.11 |
|  | SIR4/sir4∆ in a | SIR4/sir4∆ combined | 1.70 | 0.79 |
|  | SIR4/sir4∆ in alpha | SIR4/sir4∆ combined | 3.89 | 0.42 |
|  |  |  |  |  |
| Figure S3C | SIR4/SIR4 | 2 empty CEN | 13.80 | 0.01 |
|  | SIR4/SIR4 | 4 empty CEN | 2.53 | 0.64 |
|  | 2 empty CEN | 4 empty CEN | 6.14 | 0.19 |
|  |  |  |  |  |
| Figure S3E | GEV on YPR | GEV 350nM B-est | 8.21 | 0.084 |
|  | GEV on YPD | GEV on YPR | 3.57 | 0.47 |
|  | GEV 350nM B-est | GEV on YPD | 33.52 | 0.0000009 |
|  |  |  |  |  |
| Fig S4A | 2µ empty | 2µ SIR4 | 43.72 | <0.0000001 |
|  |  |  |  |  |
| Figure S4D | SIR4/SIR4 | SIR4/SIR4 + pGAL-SIR4 | 165.00 | <0.0000001 |
|  |  |  |  |  |
| Figure S7A | SIR4/SIR4 | SIR4/sir4∆ | 158.80 | <0.0000001 |
|  | SIR4/SIR4 | ubp10∆/ubp10∆ | 33.21 | 0.0000011 |
|  | SIR4/SIR4 | ubp10∆ SIR4/ubp10∆ sir4∆ | 48.64 | <0.0000001 |
|  | SIR4/sir4∆ | ubp10∆/ubp10∆ | 274.92 | <0.0000001 |
|  | SIR4/sir4∆ | ubp10∆ SIR4/ubp10∆ sir4∆ | 71.10 | <0.0000001 |
|  | ubp10∆/ubp10∆ | ubp10∆ SIR4/ubp10∆ sir4∆ | 47.50 | <0.0000001 |
|  |  |  |  |  |
| Figure S7B | SIR4/SIR4 | SIR4/sir4∆ | 158.80 | <0.0000001 |
|  | SIR4/SIR4 | yku70∆/yku70∆ | 91.02 | <0.0000001 |
|  | SIR4/SIR4 | yku70∆ SIR4/yku70∆ sir4∆ | 30.37 | 0.0000041 |
|  | SIR4/sir4∆ | yku70∆/yku70∆ | 66.41 | <0.0000001 |
|  | SIR4/sir4∆ | yku70∆ SIR4/yku70∆ sir4∆ | 17.80 | 0.0013 |
|  | yku70∆/yku70∆ | yku70∆ SIR4/yku70∆ sir4∆ | 98.90 | <0.0000001 |
|  |  |  |  |  |
| Figure S7C | SIR4/SIR4 | dot∆/dot1∆ | 39.32 | <0.0000001 |
|  | SIR4/SIR4 | ubp10∆/ubp10∆ | 33.21 | 0.0000011 |
|  | SIR4/SIR4 | dot1∆ ubp10∆/dot1∆ ubp10∆ | 111.22 | <0.0000001 |
|  | dot∆/dot1∆ | ubp10∆/ubp10∆ | 3.80 | 0.44 |
|  | dot∆/dot1∆ | dot1∆ ubp10∆/dot1∆ ubp10∆ | 3.90 | 0.42 |
|  | ubp10∆/ubp10∆ | dot1∆ ubp10∆/dot1∆ ubp10∆ | 25.60 | 0.000040 |
|  |  |  |  |  |
| Figure S7D | SIR4/SIR4 | dot∆/dot1∆ | 39.32 | <0.0000001 |
|  | SIR4/SIR4 | yku70∆/yku70∆ | 91.02 | <0.0000001 |
|  | SIR4/SIR4 | dot1∆ yku70∆/dot1∆ yku70∆ | 28.15 | 0.000012 |
|  | dot∆/dot1∆ | yku70∆/yku70∆ | 8.10 | 0.87 |
|  | dot∆/dot1∆ | dot1∆ ubp10∆/dot1∆ubp10∆ | 10.30 | 0.036 |
|  | yku70∆/yku70∆ | dot1∆ ubp10∆/dot1∆ubp10∆ | 26.30 | 0.000030 |
|  |  |  |  |  |
| Figure S7E | SIR4/SIR4 | ubp10∆/ubp10∆ | 33.21 | 0.0000011 |
|  | SIR4/SIR4 | yku70∆/yku70∆ | 91.02 | <0.0000001 |
|  | SIR4/SIR4 | yku70∆ ubp10∆/yku70∆ ubp10∆ | 24.81 | 0.000055 |
|  | ubp10∆/ubp10∆ | yku70∆/yku70∆ | 27.30 | 0.000020 |
|  | ubp10∆/ubp10∆ | yku70∆ ubp10∆/yku70∆ ubp10∆ | 8.80 | 0.066 |
|  | yku70∆/yku70∆ | yku70∆ ubp10∆/yku70∆ ubp10∆ | 10.60 | 0.03 |
|  |  |  |  |  |
| Figure S7A | SIR4/SIR4 | rif1∆/rif1∆ | 6.45 | 0.17 |
|  | SIR4/SIR4 | ku70∆ rif1∆/ku70∆ rif1∆ | 11.94 | 0.018 |
|  | SIR4/SIR4 | ku70∆ rif1∆ rif2∆/ku70∆ rif1∆ rif2∆ | 7.99 | 0.091 |
|  | rif1∆/rif1∆ | ku70∆ rif1∆/ku70∆ rif1∆ | 21.52 | 0.00025 |
|  | rif1∆/rif1∆ | ku70∆ rif1∆ rif2∆/ku70∆ rif1∆ rif2∆ | 10.61 | 0.014 |
|  | ku70∆ rif1∆/ku70∆ rif1∆ | ku70∆ rif1∆ rif2∆/ku70∆ rif1∆ rif2∆ | 5.88 | 0.21 |

* deviance = - 2 * log(likelihood ratio)
