# Supplementary material for: Competition between Heterochromatic Loci Allows the Abundance of the Silencing Protein, Sir4, to Regulate de novo Assembly of Heterochromatin
Source: PLoS Genet. 2015 Nov 20;11(11):e1005425. doi: 10.1371/journal.pgen.1005425 (PMC4654584; doi:10.1371/journal.pgen.1005425)
Supplement: S2 Table — (DOCX) [file pgen.1005425.s009.docx]

| **Strain** | **Genotype**^a^ |
| --- | --- |
| **ADR21 (W303-1a)** | ***MATa*** *ura3-1 leu2-3,112 trp1-1 his3-11,15 ade2-1 can1-100* |
| **ADR22 (W303-1b)** | ***MATα*** *ura3-1 leu2-3,112 trp1-1 his3-11,15 ade2-1 can1-100* |
| **ADR2828** | ***MATα*** *TELVII-L::URA3* |
| **ADR2830** | ***MATα*** *adh4::URA3* |
| **ADR3081 (57/2)**^b^ | ***MATa*** *his1-1* |
| **ADR3082 (m31)**^b^ | ***MATα*** *his1-1 met1* |
| **ADR3344** | ***MATa*** *sir4Δ::natMX* *[SIR4-CEN-HIS3 (pAR450)]* |
| **ADR3387** | ***MATa*** *sir4Δ::natMX* |
| **ADR3810** | ***MATa*** *SIR4-eGFP-kanMX bar1Δ* |
| **ADR4006** | ***MATa*** *bar1Δ* |
| **ADR4062** | ***MATa*** *hmrΔE::TRP1 TELVII-L::URA3* |
| **ADR4482** | ***MATa*** *hrmΔE::TRP1 TELVII-L::URA3 sir4∆::natMX* |
| **ADR4562** | ***matΔ::kanMX*** *hmlΔ::natMX hmrΔ::hphMX LYS2 ade2-1 his3-11,15::pGAL-SIR4-HIS3* |
| **ADR4564** | ***matΔ::kanMX*** *hmrΔ::hphMX* *sir3Δ::TRP1 lys2-1 ADE2* *his3-11,15::pGAL-SIR4-HIS3* |
| **ADR4592** | ***matΔ::kanMX*** *hmlΔ::natMX hmrΔ::hphMX LYS2 ade2-1 sir4Δ::Sphis5^+^* |
| **ADR4593** | ***matΔ::kanMX*** *hmrΔ::hphMX* *sir3Δ::TRP1 lys2-1 ADE2* *sir4Δ::Sphis5^+^* |
| **ADR4631** | ***matΔ::kanMX*** *hmlΔ::natMX hmrΔ::hphMX LYS2 ade2-1* *dot1Δ::Sphis5^+^* |
| **ADR4632** | ***matΔ::kanMX*** *hmrΔ::hphMX* *sir3Δ::TRP1 lys2-1 ADE2* *dot1Δ::Sphis5^+^* |
| **ADR5087** | ***matΔ::kanMX*** *hmlΔ::natMX hmrΔ::hphMX LYS2 ade2-1* *ubp10Δ::CaURA3* |
| **ADR5088** | ***matΔ::kanMX*** *hmrΔ::hphMX* *sir3Δ::TRP1 lys2-1 ADE2 ubp10Δ::CaURA3* |
| **ADR5171** | ***matΔ::kanMX*** *hmlΔ::natMX hmrΔ::hphMX LYS2 ade2-1* *dot1Δ::Sphis5^+^* *ubp10Δ::CaURA3* |
| **ADR5172** | ***matΔ::kanMX*** *hmrΔ::hphMX* *sir3Δ::TRP1 lys2-1 ADE2* *dot1Δ::Sphis5^+^* *ubp10Δ::CaURA3* |
| **ADR5389** | ***matΔ::kanMX*** *hmlΔ::natMX hmrΔ::hphMX LYS2 ade2-1* leu2-3,112::pMRP7-GAL4-ER-VP16-LEU2 *his3-11,15::pGAL-SIR4-HIS3* |
| **ADR5390** | ***matΔ::kanMX*** *hmrΔ::hphMX* *sir3Δ::TRP1 lys2-1 ADE2* leu2-3,112::pMRP7-GAL4-ER-VP16-LEU2 *his3-11,15::pGAL-SIR4-HIS3* |
| **ADR5469** | ***MATa*** *hrmΔE::TRP1 TELVII-L::URA3 sir3∆::kanMX* |
| **ADR5550** | ***matΔ::kanMX*** *hmlΔ::natMX hmrΔ::hphMX LYS2 ade2-1* *ubp10Δ::CaURA3 sir4Δ::Sphis5^+^* |
| **ADR5551** | ***matΔ::kanMX*** *hmrΔ::hphMX* *sir3Δ::TRP1 lys2-1 ADE2* *ubp10Δ::CaURA3 sir4Δ::Sphis5^+^* |
| **ADR5607** | ***matΔ::kanMX*** *hmrΔ::hphMX* *sir3Δ::TRP1 lys2-1 ADE2* *dot1Δ::CaURA3 sir4Δ::Sphis5^+^* |
| **ADR5640** | ***matΔ::kanMX*** *hmlΔ::natMX hmrΔ::hphMX LYS2 ade2-1* *dot1Δ::CaURA3 sir4Δ::Sphis5^+^* |
| **ADR5840** | ***MATa*** *hrmΔE::TRP1 TELVII-L::URA3 yku70Δ::Sphis5^+^* |
| **ADR5841** | ***matΔ::kanMX*** *hmlΔ::natMX hmrΔ::hphMX LYS2 ade2-1 yku70Δ::Sphis5^+^* |
| **ADR5842** | ***matΔ::kanMX*** *hmrΔ::hphMX* *sir3Δ::TRP1 lys2-1 ADE2* *yku70Δ::Sphis5^+^* |
| **ADR5843** | ***MATa*** *hrmΔE::TRP1 TELVII-L::URA3 ubp10Δ::Sphis5^+^* |
| **ADR5895** | ***MATa*** *hrmΔE::TRP1 TELVII-L::URA3* *dot1Δ::Sphis5^+^* |
| **ADR5920** | ***matΔ::kanMX*** *hmlΔ::natMX hmrΔ::hphMX LYS2 ade2-1 ubp10Δ::CaURA3 yku70Δ::Sphis5^+^* |
| **ADR5921** | ***matΔ::kanMX*** *hmrΔ::hphMX* *sir3Δ::TRP1 lys2-1 ADE2* *ubp10Δ::CaURA3 yku70Δ::Sphis5^+^* |
| **ADR5944** | ***matΔ::kanMX*** *hmlΔ::natMX hmrΔ::hphMX LYS2 ade2-1 dot1Δ::CaURA3 yku70Δ::Sphis5^+^* |
| **ADR5945** | ***matΔ::kanMX*** *hmrΔ::hphMX* *sir3Δ::TRP1 lys2-1 ADE2* *dot1Δ::CaURA3 yku70Δ::Sphis5^+^* |
| **ADR6181** | ***MATα*** *dot1Δ::kanMX* |
| **ADR6182** | ***MATα*** *ubp10Δ::kanMX* |
| **ADR6183** | ***MATα*** *yku70Δ::kanMX* |
| **ADR6184** | ***MATa*** *hmr∆A::ADE2* *dot1Δ::kanMX* |
| **ADR6185** | ***MATa*** *hmr∆A::ADE2* *ubp10Δ::kanMX* |
| **ADR6186** | ***MATa*** *hmr∆A::ADE2* *yku70Δ::kanMX* |
| **ADR7842** | ***matΔ::kanMX*** *hmlΔ::natMX hmrΔ::hphMX LYS2 ade2-1 sir4Δ::Sphis5^+^* ***y****ku70Δ::3HA-KlTRP1* |
| **ADR7846** | ***matΔ::kanMX*** *hmrΔ::hphMX* *sir3Δ::TRP1 lys2-1 ADE2* *sir4∆::CaURA3* *yku70Δ::natMX* |
| **ADR7962** | ***matΔ::kanMX*** *hmlΔ::natMX hmrΔ::hphMX LYS2 ade2-1* *rif1Δ::Sphis5^+^* |
| **ADR7966** | ***matΔ::kanMX*** *hmrΔ::hphMX* *sir3Δ::TRP1 lys2-1 ADE2* *rif1Δ::Sphis5^+^* |
| **ADR7972** | ***matΔ::kanMX*** *hmlΔ::natMX hmrΔ::hphMX LYS2 ade2-1* ***y****ku70Δ::3HA-KlTRP1 rif1Δ::Sphis5^+^* |
| **ADR7975** | ***matΔ::kanMX*** *hmrΔ::hphMX* *sir3Δ::TRP1 lys2-1 ADE2* ***y****ku70Δ::natMX rif1Δ::Sphis5^+^* |
| **ADR7986** | ***matΔ::kanMX*** *hmlΔ::natMX hmrΔ::hphMX LYS2 ade2-1* ***y****ku70Δ::3HA-KlTRP1 rif1Δ::Sphis5^+^* *rif2∆::CaURA3* |
| **ADR7989** | ***matΔ::kanMX*** *hmrΔ::hphMX* *sir3Δ::TRP1 lys2-1 ADE2* ***y****ku70Δ::natMX rif1Δ::Sphis5^+^* *rif2∆::CaURA3* |
| **ADR8824** | ***MATα*** *rif1∆::hphMX* |
| **ADR8833** | ***MATα*** *rif1∆::hphMX rif2∆::natMX* |
| **ADR8888** | ***matΔ::kanMX*** *hmrΔ::hphMX* *sir3Δ::TRP1 lys2-1 ADE2 ubp10∆::CaURA3 rif1∆::Sphis5^+^ rif2∆::natMX* |
| **ADR8901** | ***MATa*** *yku70Δ::Sphis5^+^ rif1∆::hphMX hmrΔE::TRP1 TELVII-L::URA3* |
| **ADR8903** | ***MATa*** *yku70Δ::Sphis5^+^ rif2∆::natMX hmrΔE::TRP1 TELVII-L::URA3* |
| **ADR8907** | ***MATa*** *yku70Δ::Sphis5^+^ rif1∆::hphMX rif2∆::natMX hmrΔE::TRP1 TELVII-L::URA3* |
| **ADR8913** | ***matΔ::kanMX*** *hmrΔ::hphMX* *sir3Δ::TRP1 lys2-1 ADE2 dot1∆::natMX rif1∆::CaURA3 rif2∆::Sphis5^+^* |
| **ADR8916** | ***matΔ::kanMX*** *hmlΔ::natMX hmrΔ::hphMX LYS2 ade2-1 dot1∆::3HA-KlTRP1 rif1∆::CaURA3 rif2∆::Sphis5^+^* |
| **ADR8922** | ***matΔ::kanMX*** *hmlΔ::natMX hmrΔ::hphMX LYS2 ade2-1 ubp10∆::CaURA3 rif1∆::Sphis5^+^ rif2∆::3HA-KlTRP1* |
| **ADR8936** | ***MATa*** *dot1Δ::Sphis5^+^ rif1∆::hphMX hmrΔE::TRP1 TELVII-L::URA3* |
| **ADR8939** | ***MATa*** *dot1Δ::Sphis5^+^ rif2∆::natMX hmrΔE::TRP1 TELVII-L::URA3* |
| **ADR8944** | ***MATa*** *dot1Δ::Sphis5^+^ rif1∆::hphMX rif2∆::natMX hmrΔE::TRP1 TELVII-L::URA3* |
| **ADR8953** | ***MATa*** *rif2∆::natMX hmrΔE::TRP1 TELVII-L::URA3* |
| **ADR8958** | ***MATa*** *ubp10Δ::Sphis5^+^ rif1∆::hphMX hmrΔE::TRP1 TELVII-L::URA3* |
| **ADR8961** | ***MATα*** *ubp10Δ::Sphis5^+^ rif2∆::natMX hmrΔE::TRP1 TELVII-L::URA3* |
| **ADR8962** | ***MATa*** *ubp10Δ::Sphis5^+^ rif1∆::hphMX rif2∆::natMX hmrΔE::TRP1 TELVII-L::URA3* |
| **ADR8969** | ***MATa*** *rif1∆::hphMX hmrΔE::TRP1 TELVII-L::URA3* |
| **ADR8974** | ***MATa*** *rif1∆::hphMX rif2∆::natMX hmrΔE::TRP1 TELVII-L::URA3* |
| **ADR9190** | ***matΔ::kanMX*** *hmlΔ::natMX hmrΔ::hphMX LYS2 ade2-1 rif1∆::CaURA3 rif2∆::Sphis5^+^* |
| **ADR9196** | ***matΔ::kanMX*** *hmrΔ::hphMX* *sir3Δ::TRP1 lys2-1 ADE2 rif1∆::hphMX rif2∆::natMX* |
| **GCY310**^c^ | ***MATa*** *hmr∆A::ADE2 esc8Δ::kanMX* |
| **GCY317**^c^ | ***MATa*** *hmr∆A::ADE2* |
| **JRY8828**^d^ | ***matΔ::kanMX*** *hmlΔ::natMX hmrΔ::hphMX LYS2 ade2-1* |
| **JRY8829**^d^ | ***matΔ::kanMX*** *hmrΔ::hphMX* *sir3Δ::TRP1 lys2-1 ADE2* |

^a^ All strains are isogenic to W303-1a (ADR21) except ADR3081 and ADR3082

^b^ Kindly provided by Fred Winston, Harvard Medical School, Boston, MA

^c^ Kindly provided by David Shore, University of Geneva, Switzerland

^d^ Kindly provided by Erin Osborne and Jasper Rine, UC, Berkeley, CA
